# Linear and Star‐Shaped Extended Di‐ and Tristyrylbenzenes: Synthesis, Characterization and Optical Response to Acid and Metal Ions

**DOI:** 10.1002/chem.202000893

**Published:** 2020-06-08

**Authors:** Hao Zhang, Eugen A. Kotlear, Soh Kushida, Steffen Maier, Frank Rominger, Jan Freudenberg, Uwe H. F. Bunz

**Affiliations:** ^1^ Organisch-Chemisches Institut Ruprecht-Karls-Universität Heidelberg Im Neuenheimer Feld 270 69120 Heidelberg Germany

**Keywords:** acidochromicity, metal ions, optical response, sensors, tristyrylbenzene

## Abstract

Two linear 1,4‐distyrylbenzenes and five star‐shaped 1,3,5‐tristyrylbenzene derivatives (**L_2a_** and **L_2b_**, **Y_0_–Y_3_** and **Y_NBu_**) were synthesized and spectroscopically characterized. The photophysical properties, optical response to acid and metal ions were investigated. Upon backbone extension of linear distyrylbenzenes or the introduction of dibutylanilines, the electronic spectra are redshifted. Incorporation of electron‐deficient pyridyl units does not significantly affect the optical properties. Variation of the number of pyridine rings and substitution pattern tune the fluorescence response to acids and metal ions. The novel arenes discriminate Al^3+^, Mn^2+^, Fe^3+^, Fe^2+^, Cd^2+^, Ag^+^ and Hg^2+^.

## Graphical abstract

**Make a star**! A series of extended star‐shaped tristyrylbenzenes has been developed. Their optoelectronic properties can be tuned through the aryl terminus, which makes them useful sensors for protons or metal ions (see figure).

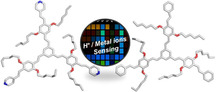

## Introduction

Functional π‐conjugated chromophores with linear^1^ or star‐shaped geometry^2^ are relevant responsive materials.^3^ 1,3,5‐Tristyrylbenzene^4^ is an excellent core for dendritic molecules.2c, 5 Peripheral and core substitution allow tuning of photoluminescence^6^ and mesogenic behavior.2b

Manipulation of the substituents in X‐shaped molecules leads to disjunct frontier molecular orbitals and tunable optical properties.^7^ If pyridines or dialkylanilines are incorporated, they become acidochromic.^8^ Both size and symmetry of the conjugated system affect the properties and performance in applications.5c, 9 We describe the synthesis, photophysical characterization, and acid/metal ion response of two linear 1,4‐distyryl (**L_2a_** and **L_2b_**) and five star‐shaped styrylbenzene derivatives (**Y_0_–Y_3_** and **Y_NBu_**).

## Results and Discussion

### Synthesis, X‐ray crystallographic analyses and liquid crystalline behavior

**Scheme 1:**
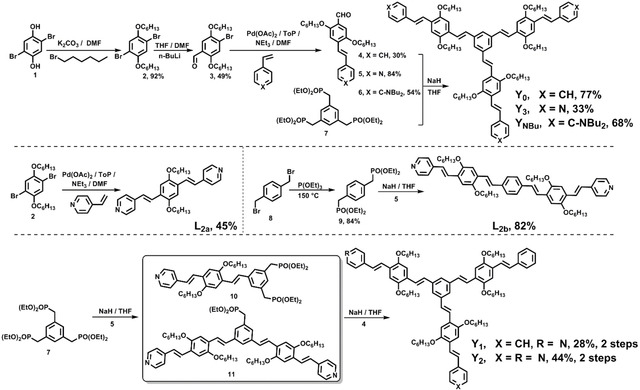
Synthetic route to linear 1,4‐distyrylbenzenes (**L_2a_–L_2b_**) and star‐shaped 1,3,5‐tristyrylbenzene derivatives (**Y_0_–Y_3_** and **Y_NBu_**).

**L_2a_** is a distyrylbenzene substituted with two pyridines. **L_2b_** was designed as an analogue of **L_2a_** with a longer effective conjugation length.^10^ **Y_0_** is a star‐shaped and *C*_3_‐symmetric π‐system bearing three identical arms. Based on this known skeleton (**Y_0_**) reported by Maier et al.,5c **Y_1_**_–**3**_ and **Y_NBu_** are obtained either by variation of the number of pyridine rings or the introduction of electron‐rich dibutylaniline groups, respectively (Scheme 1).

Compound **1** was reacted with 1‐bromohexane (K_2_CO_3_, anhydrous DMF) to afford **2**, which was transformed by a Bouveault reaction to the monoaldehyde **3**. Heck coupling between **3** and three arylethylenes produced **4**–**6**, which were in turn subjected to a Horner reaction with triphosphonate **7** in the presence of sodium hydride, thus, affording **Y_0_** (77 %), **Y_3_** (33 %) and **Y_NBu_** (68 %). As a reference system, distyrylbenzene **L_2a_** was obtained after Heck reaction of **2** and 4‐vinylpyridine (45 %). To obtain π‐extended **L_2b_**, **8** was subjected to an Arbuzow reaction with triethyl phosphite, followed by a Horner reaction with monoaldehyde **5. L_2b_** was isolated after column chromatography on silica gel (82 %). **Y_1_** and **Y_2_** were obtained via two one‐pot procedures starting from **7** with aldehydes **5** and **4**. For **Y_1_**, the first Horner reaction of **7** with monoaldehyde **5** (molar ratio: 1:1.1) afforded **10**, which was subjected to a second Wittig–Horner reaction with **4** in situ (28 % over two steps). By changing the stoichiometry of **7** and **5** (1:2.2), **Y_2_** was isolated by a similar two‐step routine with a yield of 44 %. It should be noted that potassium *tert*‐butoxide as a base did not work.^11^ **L_2a_/L_2b_** and **Y_0_–Y_3_** are yellow or orange solids, while **Y_NBu_** is a yellow oil. All styrylbenzene derivatives (SBs) are well soluble in common organic solvents.

**Figure 1:**
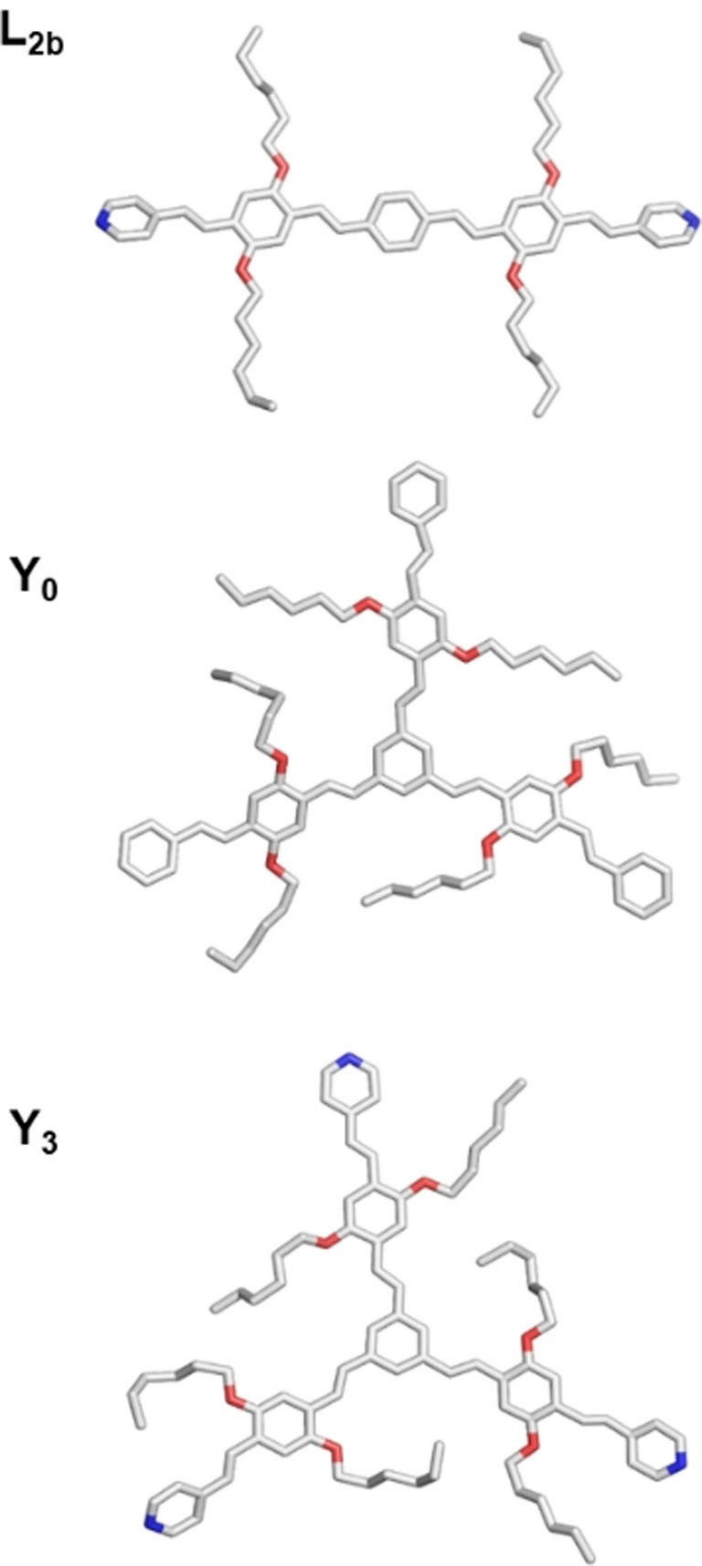
Single‐crystal structures of **L_2b_**, **Y_0_** and **Y_3_**.

We obtained single crystals for **Y_0_** (from DCM/methanol), **L_2b_** and **Y_3_** (from DCM/*n*‐hexane) and performed X‐ray analysis. Figure 1 depicts the structure and Table S1–S3 summarize the corresponding data. As shown in Figure S1, all derivatives are almost planar and their vinylic linkers display *E*‐configuration. However, the packing patterns of **L_2b_**, **Y_0_** and **Y_3_** deviate significantly from each other. **L_2b_** packs in parallel layer stacks with some displacement. **Y_0_** displays a 2‐dimensional wall‐like arrangement and the orientation of molecular planes in adjacent parallel walls is different. In **Y_3_**, a pattern of parallel planes was observed, which extends in three directions.

Similar to Maier's star‐shaped compounds, nematic liquid crystalline phases from isotropic during cooling scans were observed for **Y_0_**_–**2**_ as investigated by temperature‐dependent polarization optical micrographs (Figure S2).5c No liquid crystalline behaviour was detected for oily **Y_NBu_** at room temperature.

### Photophysical properties and theoretical calculations

**Figure 2:**
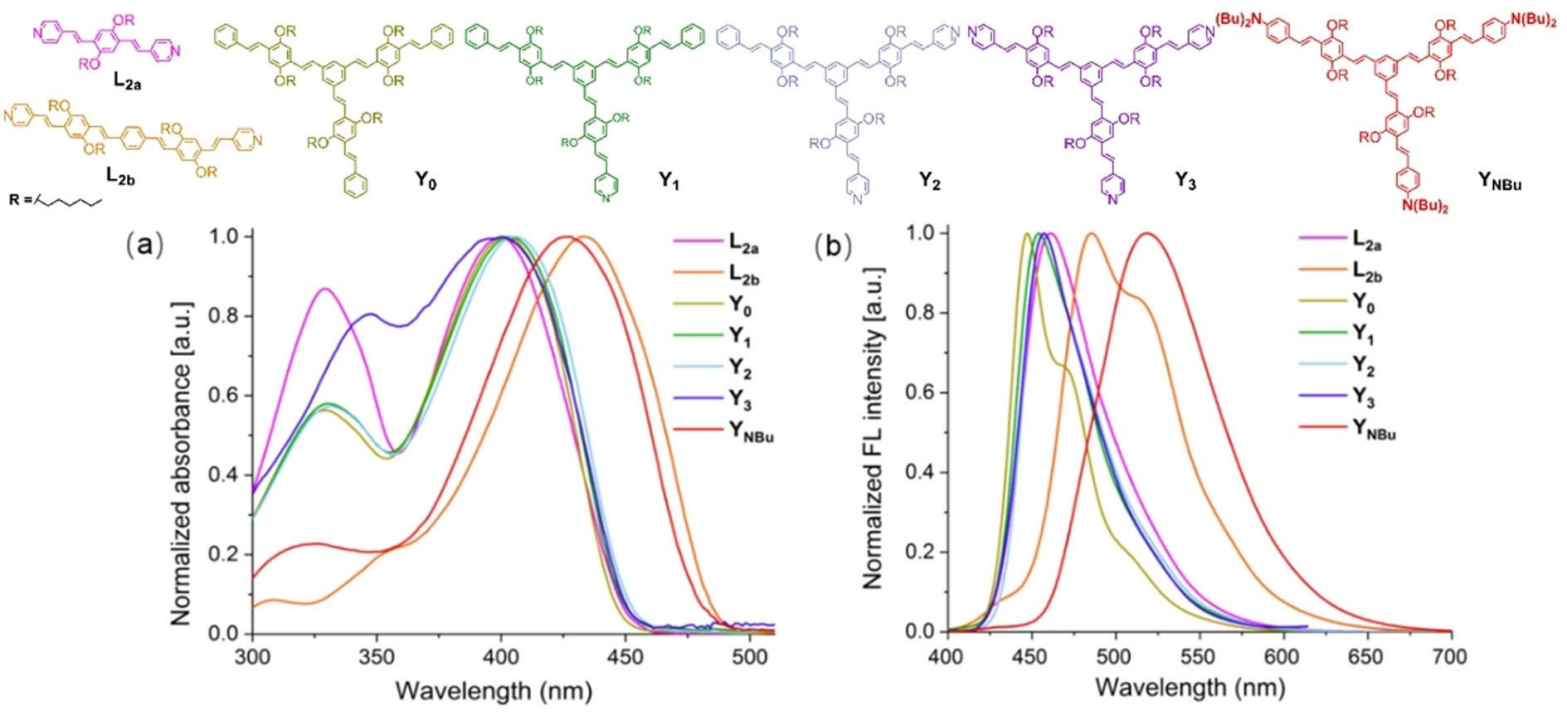
Normalized UV/vis absorption (a) and emission (b) spectra of SBs in THF.

The normalized absorption and emission spectra of the compounds in dilute THF are shown in Figure 2. Table 1 summarizes the photophysical data. Due to the *meta*‐conjugation, absorption spectra of star‐shaped series **Y_0_‐Y_3_** are superimposable to that of **L_2a_**, with a maximum absorption centered at around 400 nm and a shoulder peak located at 326–348 nm. It is mainly attributed to the extension of conjugation or the electron‐rich dibutylaniline groups.

**Table 1:** Photophysical data (in THF) and calculated energy gaps for SBs.

Compds.	*λ*_abs_ [nm]^[a]^	*λ*_max,em_ [nm]	QY^[b]^	*τ*_f_ [ns]	*λ*_st_ [nm]/*ν*_st_ [cm^−1^]^[c]^	Calcd energy gap [eV, nm]
**L_2a_**	329 (2.37), 400 (2.88)	461	0.64	2.00	61, 3308	3.06, 405
**L_2b_**	432 (7.31)	485	0.68	1.10	53, 2529	2.56, 484
**Y_0_**	329 (6.17), 401 (11.0)	447	0.82	1.39	46, 2566	2.97, 417
**Y_1_**	330 (5.68), 403 (9.89)	454	0.73	1.51	51, 2787	2.86, 433
**Y_2_**	331 (7.13), 405 (12.4)	456	0.72	1.52	51, 2761	2.87, 432
**Y_3_**	348 (2.29), 401 (2.86)	457	0.79	1.67	56, 3118	2.96, 418
**Y_NBu_**	326 (1.91), 427 (8.64)	519	0.61	1.41	92, 4151	2.82, 440

[a] Measured in THF and extinction coefficients (*ϵ*_max_×10^4^ m ^−1^ **⋅**cm^−1^) are shown in parentheses. [b] QY=quantum yield. [c] *ν*_st_=1/*λ*_abs_−1/*λ*_em_.

In THF, the trend in the emission maxima (Figure 2 b) follows the order **Y_NBu_**>**L_2b_**>**L_2a_**>**Y_3_**>**Y_2_**>**Y_1_**>**Y_0_**. **L_2b_** exhibits a redshifted green emission with a vibronic structure relative to that of **L_2_** **a**, owing to its longer conjugation length. Asymmetric **Y_1_** and **Y_2_** show similar emission maxima in comparison to symmetric **Y_3_** while their maxima are redshifted by ca. 10 nm compared to symmetric hydrocarbon **Y_0._** The fluorescence maximum of **Y_NBu_** bathochromically shifted to 519 nm (Stokes shift of 4151 cm^−1^). The large Stokes shift observed for **Y_NBu_** is caused by its higher dipole moment in its excited state stabilized in more polar solvents.^12^ **L_2a_**, **L_2b_** and **Y_0_**_–**3**_ display quantum yields (*Φ*_f_) varying from 0.64 to 0.82, while **Y_NBu_** exhibits a green fluorescence with a quantum yield of 0.61. Although **Y_0_**_–**3**_ and **Y_4_** have similar conjugation skeletons, dibutylamine‐containing **Y_4_** shows a lower quantum yield, which might be attributed to the free intramolecular rotation or the existence of photoelectron transfer facilitated by the flexible dibutylamine groups.^13^

DFT calculations (B3LYP/6‐31++G**)^14^ provide further insight into optoelectronics. The calculated HOMO–LUMO energy gaps for **L_2a_/L_2b_, Y_0_–Y_3_** and **Y_NBu_** are in the range from 2.56 to 3.06 eV (Figure S4). The lower gaps of **Y_NBu_** or **L_2b_** are a result of the extended conjugation, resulting in distinct bathochromic shifts in the electronic absorption spectra. These results, as well as the trend of absorption maxima calculated by TDDFT methods (Figure S3) are consistent with the experiment.

### Fluorochromicity

**Figure 3:**
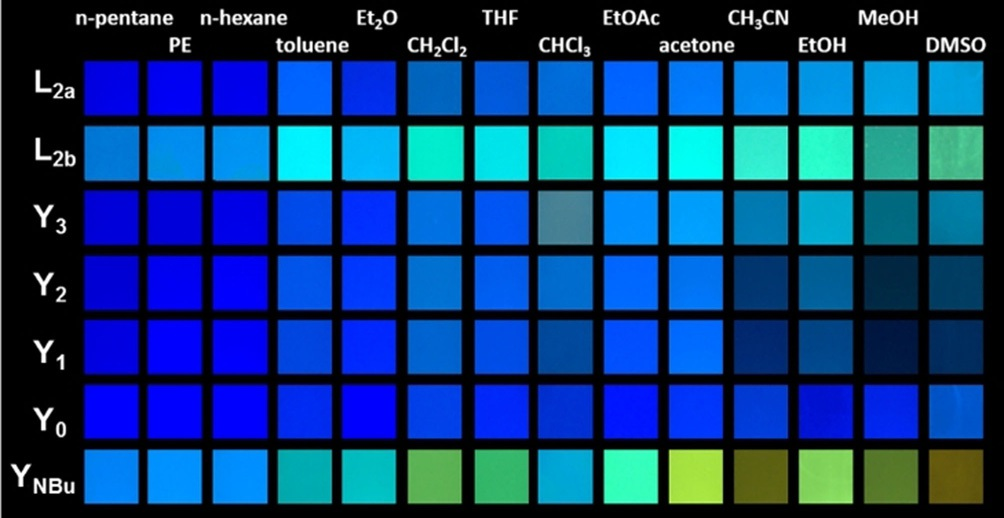
Photograph of SBs in different solvents under a hand‐held black light with illumination at 365 nm.

Figure 3 shows the emission behaviors of SBs in solvents with different polarity. Negligible changes are observed in the emission color of **L_2a_** and **Y_1_**_–**3**_ with pyridine rings. **L_2b_** and **Y_NBu_** emit redshifted with increasing solvent polarity.^12^ The effect of solvents on the emission features was evaluated by the Lippert–Mataga plot^15^ (Supporting Information). All of the SBs displayed linear dependence of Δν on Δf together with different slopes, which was largest for **L_2b_** and **Y_NBu_** (Figure S5 and Table S5). The slope of the fitting line for **Y_NBu_** is the highest, up to 9673 cm^−1^, comparable to that of the X‐shaped distyrylbenzenes,^16^ which further indicated its larger dipole moment changes between the ground and excited states (μ_e_–μ_g_), leading to the pronounced solvent sensitivity.^17^

### Optical response to protons

**Figure 4:**
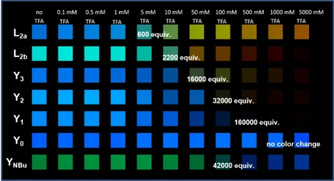
Photograph of SBs in THF (*c*=4 μg mL^−1^) with increasing content of TFA under a hand‐held black light with illumination at 365 nm (inset data: the number of equivalents of TFA).

Pyridines or dibutylanilines are basic. All SBs (except **Y_0_**) display an acidochromic change of their absorption and emission spectra (Figure 4). SBs with pyridine units experience a redshift, accompanied with a loss of fluorescence intensity. Upon protonation the donor‐acceptor character of these SBs increases and therefore internal charge transfer is favored. The addition of TFA to **Y_NBu_** leads to strongly blue emissive species, in which the dibutylamino groups are protonated. The HOMO is stabilized by protonation, resulting in an increased HOMO–LUMO gap. The color change is detected by eye. As expected, **Y_3_** is more sensitive to protonation compared to **Y_1_** and **Y_2_**, as there are three pyridine rings as interaction sites.

**Figure 5:**
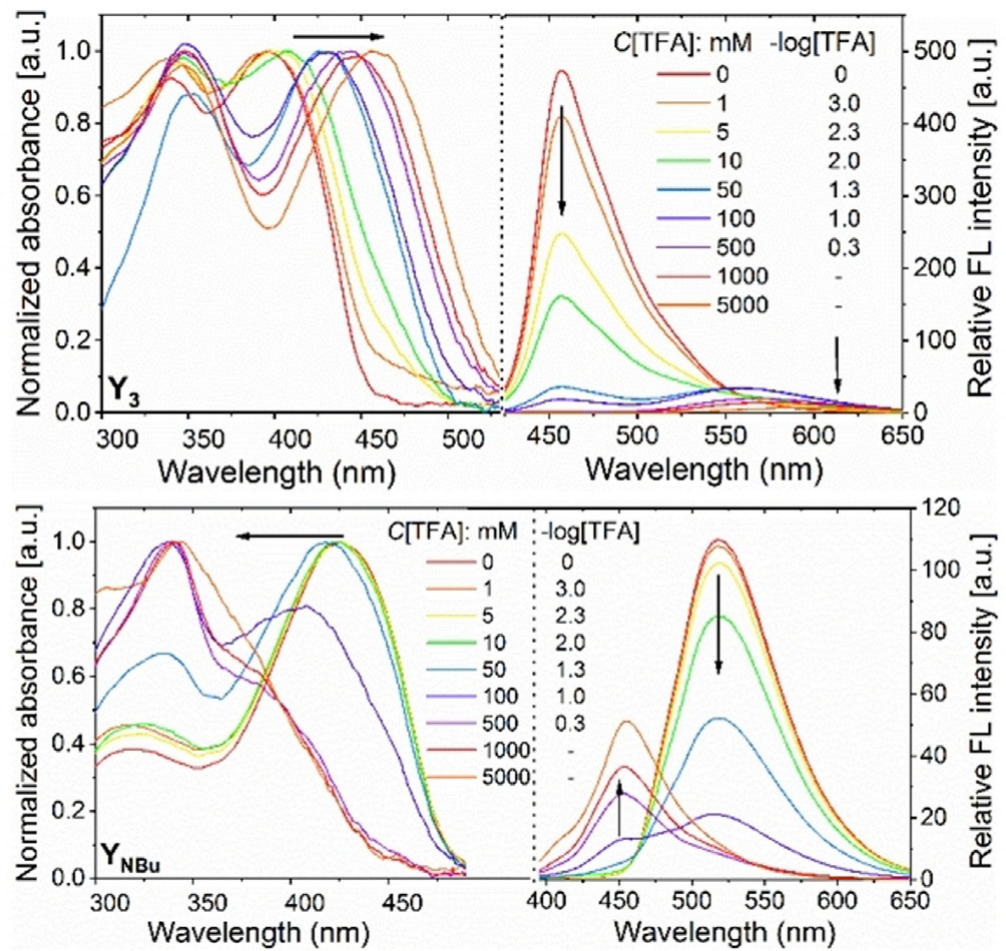
Normalized UV/vis absorption (left) and emission (right) spectra for the titrations of **Y_3_** (top) and **Y_NBu_** (bottom) in THF with different concentration of TFA.

As shown in Figure 5, when ‐log [TFA] of approx. 1.30 is reached (*c*=50 mm), **Y_3_** shows a redshifted absorption (30 nm) and a 100 nm redshift and distinct attenuation of its emission. With excess acid (*c*=500 mm, ‐log [TFA]<1.0), the original absorption band of **Y_NBu_** at around 427 nm diminished and a new distinct band formed at 338 nm. A blueshift in emission of Δ*λ*=−69 nm was observed.

### Optical response to metal ions

**Figure 6:**
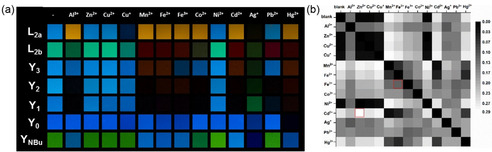
(a) Exposure of all the SBs (10 μm) to different metal cations in DCM. Photographs were taken under a hand‐held black light (365 nm) with a digital camera; (b) Autocorrelation plot (RAW rg values) of all the SBs in DCM after exposure to metal ions.

Dilute solutions of all the SBs in DCM were exposed to an excess of salts of 13 cations (Al^3+^, Zn^2+^, Cu^2+^, Cu^+^, Mn^2+^, Fe^2+^, Fe^3+^, Co^2+^, Ni^2+^, Cd^2+^, Ag^+^, Pb^2+^ and Hg^2+^, added as perchlorates, and CuI) (Figure 6 a). Only Hg^2+^ quenches luminescence of **Y_0_**. Except for Zn^2+^, Cu^2+^, Ni^2+^, the addition of the remaining ten metal ions leads to either quenching or a redshift in emission of SBs with pyridine units (**L_2a_**, **L_2b_** and **Y_1_**_–**3**_). In the case of **Y_NBu_**, Al^3+^, Mn^2+^, Fe^3+^, Fe^2+^, Cd^2+^, Ag^+^, and Hg^2+^ induce a blueshift in emission, as expected for a coordination at the aniline nitrogen. Al^3+^, Mn^2+^, Cd^2+^ and Co^2+^ are quenchers for **Y_1_** and **Y_2_**, but they less impact photoluminescence of **Y_3_**. Al^3+^, Mn^2+^, Fe^3+^, Fe^2+^, Cd^2+^, Ag^+^ and Hg^2+^ can be easily distinguished from each other through the responses of the SBs by the naked eye. It is challenging to discriminate Cd^2+^ from Zn^2+^.^18^ SBs with pyridine or dibutylaniline groups display fluorescence responses in the presence of Cd^2+^ but no response with Zn^2+^.

(1) $$\sigma_{m,\;n}\left(r,g\right)=\sqrt{\frac{\sum_{SBs1}^{SBs7}\left(r_{n}-r_{m}\right)2+\left(g_{n}-g_{m}\right)2}{3{}^{*}7}}$$

Statistical evaluation of differences in emission colors after exposure to metal ions was performed. Figure 6 b shows the autocorrelation plot of their response. The brightness independent color coordinates rg of the RAW data of the photographs were determined and treated with MANOVA statistics (Eq. (1)).7c, 19 Zn^2+^, Cu^2+^ and Ni^2+^ are hard to discern due to their weak coordination and thus have similar color responses. All of the other investigated metal ions are distinguished.

## Conclusions

We have synthesized linear 1,4‐distyryl and star‐shaped 1,3,5‐tristyrylbenzene derivatives (**L_2a_**/**L_2b_**, **Y_0_**–**Y_3_** and **Y_NBu_**). These are strongly fluorescent in dilute solutions. **Y_NBu_** works as polarity sensor due to its response to different solvents. Upon protonation, all of the pyridine‐containing compounds, **L_2a_**/**L_2b_** and **Y_0_–Y_3_**, show a pronounced redshift, and the fluorophore with dibutylaniline groups **Y_NBu_** displays a blueshift in emission. Ten metal ions such as Al^3+^, Mn^2+^, Fe^3+^, Fe^2+^, Cd^2+^, Ag^+^ and Hg^2+^ were well discriminated.

## Experimental Section

### General procedure 1 (GP1)

Synthesis of intermediates **4**–**6** by Heck reaction. The reaction was performed in a heat‐gun‐dried 50 mL Schlenk tube under a nitrogen atmosphere. The brominated intermediate (1.0 equiv) and the vinyl compound (1.14 equiv) were dissolved in dry DMF. Pd(OAc)_2_ (5 mol %), tris(*o*‐tolyl)phosphine (0.1 equiv) and dry triethylamine (0.8 mL) were added and the mixture was stirred at 110 °C for 48 h. After the reaction mixture was cooled to ambient temperature, it was poured into water to give a suspension which was extracted with DCM. The combined organic layers were washed with brine, dried over MgSO_4_ and the solvents were removed under reduced pressure. The residues were purified by column chromatography.

### General procedure 2 (GP2)

Synthesis of symmetric **Y_0_, Y_3_** and **Y_NBu_** by Wittig–Horner reaction. Under nitrogen atmosphere, the triphosphonate **7** (1.0 equiv) was dissolved in dry THF and the solution was cooled to 0 °C. NaH (15.0 equiv) was added carefully and the mixture was stirred at 0 °C for 40 min before the monoaldehyde **4**–**6** (4.5 equiv) was added slowly. The reaction mixture was then allowed to warm to RT and further stirred for 3 days. After removing THF on a rotary evaporator, the residues were purified on silica gel column.

### 4,4′‐((1*E*,1′*E*)‐(2,5‐Bis(hexyloxy)‐1,4‐phenylene)bis(ethene‐2,1‐diyl))dipyridine (L_2a_)

Under nitrogen atmosphere, a solution of **2** (218 mg, 500 μmol), 4‐vinylpyridine (118 mg, 1.20 mmol), Pd(OAc)_2_ (11.2 mg, 50 μmol), tris(*o*‐tolyl)phosphine (30.4 mg, 100 μmol) and triethylamine (1.20 mL) in dry DMF (10 mL) was stirred at 110 °C for 48 h. After the reaction mixture was cooled to ambient temperature, it was poured into water to give a suspension which was extracted with DCM. The combined organic layers were washed with brine dried over MgSO_4_ and the solvents were removed under reduced pressure. The residues were purified by column chromatography (silica gel, DCM/EE=5:1, *R*_f_=0.21) and afforded **L_2a_** as a yellow solid (110 mg, 225 μmol, 45 %). M.p.: 135–136 °C. ^1^H NMR (300 MHz, CDCl_3_) *δ* 8.58 (d, *J=*6.0 Hz, 4 H), 7.70 (s, 1 H), 7.64 (s, 1 H), 7.38 (d, *J=*6.0 Hz, 4 H), 7.18–7.08 (m, 3 H), 7.05 (s, 1 H), 4.08 (t, *J=*6.5 Hz, 4 H), 1.95–1.82 (m, 4 H), 1.63–1.49 (m, 4 H), 1.47–1.33 (m, 8 H), 0.93 (t, *J=*7.0 Hz, 6 H). ^13^C NMR (100 MHz, CDCl_3_) *δ* 151.7, 150.1, 149.8, 145.4, 129.7, 128.3, 128.2, 127.6, 126.9, 126.7, 126.5, 126.0, 123.6, 121.0, 114.6, 114.1, 111.2, 110.9, 69.7, 31.8, 29.5, 26.1, 22.8, 14.2. IR (cm^−1^): 2942, 2915, 2360, 1592, 1476, 1209, 1028, 968, 850, 803, 596, 525. HRMS (MALDI): *m*/*z* [*M*+H]^+^ calcd for C_32_H_41_N_2_O_2_ 485.3168, found 485.372.

### 2,5‐Bis(hexyloxy)‐4‐(2‐(pyridin‐4‐yl)vinyl)benzaldehyde (5)

According to **GP1** a solution of monoaldehyde **3** (385 mg, 1.00 mmol), 4‐vinylpyridine (214 mg, 1.14 mmol), Pd(OAc)_2_ (11.2 mg, 50.0 μmol), tris(*o*‐tolyl)phosphine (30.4 mg, 100 μmol) and triethylamine (0.7 mL) in DMF (10 mL) was stirred at 110 °C for 48 h. Column chromatography (silica gel, PE/EA=5:1, *R*_f_=0.20) afforded **5** as a yellow viscous oil (343 mg, 840 μmol, 84 %). ^1^H NMR (300 MHz, CDCl_3_) *δ* 10.59 (s, 1 H), 8.73 (d, *J=*5.2 Hz, 2 H), 7.78 (d, *J=*16.5 Hz, 1 H), 7.52 (d, *J=*5.6 Hz, 2 H), 7.47 (s, 1 H), 7.30 (t, *J=*8.2 Hz, 2 H), 4.24 (t, *J=*6.5 Hz, 2 H), 4.16 (t, *J=*6.5 Hz, 2 H), 2.11–1.90 (m, 4 H), 1.72–1.27 (m, 14 H), 1.05 (dd, *J=*6.4, 4.4 Hz, 6 H). ^13^C NMR (125 MHz, CDCl_3_) *δ* 189.1, 156.0, 151.1, 150.2, 144.7, 132.7, 129.4, 127.6, 125.1, 121.1, 111.3, 110.3, 69.3, 69.2, 31.6, 29.2, 25.9, 22.7, 14.1. IR (cm^−1^): 2928, 2857, 1676, 1601, 1422, 1206, 1018, 529. HRMS (DART): *m*/*z* [*M*+H]^+^ calcd for C_26_H_36_NO_3_ 410.2690, found 410.2687.

### 4‐(4‐(Dibutylamino)styryl)‐2,5‐bis(hexyloxy)benzaldehyde (6)

According to **GP1** a solution of monoaldehyde **3** (771 mg, 2.00 mmol), 4‐dibutylaminostyrene (509 mg, 2.20 mmol), Pd(OAc)_2_ (22.4 mg, 100 μmol), tris(*o*‐tolyl)phosphine (60.9 mg, 200 μmol) and triethylamine (1.5 mL) in DMF (20 mL) was stirred at 110 °C for 48 h. Column chromatography (silica gel, PE/EA=20:1, *R*_f_=0.50) afforded **6** as a yellow viscous oil (582 mg, 1.10 mmol, 54 %). ^1^H NMR (400 MHz, CDCl_3_) *δ* 10.42 (s, 1 H), 7.41 (d, *J=*8.7 Hz, 1 H), 7.32–7.27 (m, 1 H), 7.25–7.03 (m, 3 H), 6.67–6.40 (m, 3 H), 4.17–3.93 (m, 4 H), 3.39–3.20 (m, 4 H), 1.91–1.74 (m, 4 H), 1.63–1.47 (m, 8 H), 1.42–1.26 (m, 12 H), 0.92 (m, 12 H). ^13^C NMR (100 MHz, CDCl_3_) *δ* 189.4, 189.2, 156.6, 155.8, 151.1, 150.5, 148.5, 147.7, 136.2, 135.9, 132.9, 132.8, 130.5, 128.5, 124.5, 123.9, 123.7, 123.4, 120.7, 117.6, 114.8, 111.7, 111.1, 110.2, 109.9, 109.7, 69.4, 69.3, 50.9, 31.7, 29.7, 29.4, 26.0, 22.8, 20.5, 14.2, 14.1. IR (cm^−1^): 2955, 2928, 2870, 1682, 1612, 1519, 1466, 1366, 1214, 1186, 811. HRMS (DART): *m*/*z* [*M*+H]^+^ calcd for C_35_H_54_NO_3_ 536.4098, found 536.4101.

### 1,3,5‐Tris((*E*)‐2,5‐bis(hexyloxy)‐4‐((*E*)‐styryl)styryl)benzene (Y_0_)

According to **GP2** a solution of triphosphonate **7** (42.3 mg, 80.0 μmol) in dry THF (8 mL) was treated with NaH (28.8 mg, 1.20 mmol) and monoaldehyde **4** (105 mg, 256 μmol) was added. The crude product was purified by column chromatography (silica gel, PE/EA=20:1, *R*_f_=0.41) to yield the desired compound **Y_0_** as a yellow green powder (79.6 mg, 61.6 μmol, 77 %). M.p.: 110–111 °C. ^1^H NMR (600 MHz, CDCl_3_) *δ* 7.66–7.49 (m, 15 H), 7.39 (t, *J=*7.6 Hz, 6 H), 7.32–7.22 (m, 6 H), 7.22–7.14 (m, 9 H), 4.11 (dd, *J=*15.1, 6.6 Hz, 12 H), 1.99–1.85 (m, 12 H), 1.59 (td, *J=*13.5, 7.0 Hz, 12 H), 1.48–1.34 (m, 24 H), 0.93 (dt, *J=*25.7, 6.8 Hz, 18 H). ^13^C NMR (150 MHz, CDCl_3_) *δ* 151.3, 151.2, 138.7, 138.1, 129.0, 128.9, 128.7, 127.5, 127.1, 126.6, 124.1, 123.6, 111.0, 110.8, 69.7, 31.7, 29.5, 26.1, 22.8, 14.2. IR (cm^−1^): 2926, 2856, 2360, 1419, 1199, 958, 750, 690, 598, 506. HRMS (MALDI): *m*/*z* [M]^+^ calcd for C_91_H_114_O_6_ 1290.8615, found 1290.947.

### 1,3,5‐Tris((*E*)‐2,5‐bis(hexyloxy)‐4‐((*E*)‐2‐(pyridin‐4‐yl)vinyl)styryl)benzene (Y_3_)

According to **GP2** a solution of triphosphonate **7** (42.3 mg, 80.0 μmol) in dry THF (8 mL) was treated with NaH (28.8 mg, 1.20 mmol) and monoaldehyde **5** (114.7 mg, 280 μmol) was added. The crude product was purified by column chromatography (silica gel, DCM/MeOH=50:1, *R*_f_=0.35) to yield the desired compound **Y_3_** as an orange solid (34.9 mg, 26.9 μmol, 33 %). M.p.: 122–123 °C. ^1^H NMR (600 MHz, CDCl_3_) *δ* 8.58–8.50 (d, *J=*5.4 Hz, 6 H), 7.71–7.39 (m, 12 H), 7.23–6.63 (m, 15 H), 4.01–3.93 (m, 12 H), 1.91–1.83 (m, 12 H), 1.58 (m, 12 H), 1.39–1.27 (m, 24 H), 0.96–0.89 (m, 18 H). ^13^C NMR (150 MHz, CDCl_3_) *δ* 151.9, 151.5, 151.4, 150.8, 150.4, 149.9, 145.6, 139.0, 138.8, 130.4, 130.3, 129.9, 128.5, 128.3, 126.3, 126.2, 125.7, 125.6, 124.6 124.3, 124.2, 121.1, 115.9, 111.3, 111.0, 109.6, 69.9, 69.7, 68.8, 32.2, 31.9, 29.9, 29.7, 26.3, 26.2, 22.9, 14.4. IR (cm^−1^): 2925, 2856, 2320, 1591, 1498, 1416, 1204, 966, 803, 517. HRMS (MALDI): *m*/*z* [*M*+H]^+^ calcd for C_87_H_112_N_3_O_6_ 1295.8629, found 1295.8624.

### 4,4′,4′′‐((1*E*,1′*E*,1′′E)‐(((1*E*,1′*E*,1′′*E*)‐Benzene‐1,3,5‐triyltris(ethene‐2,1‐diyl))tris(2,5‐bis(hexyloxy)benzene‐4,1‐diyl))tris(ethene‐2,1‐diyl))tris(*N*,*N*‐dibutylaniline) (Y_NBu_)

According to **GP2** a solution of triphosphonate **7** (42.3 mg, 80.0 μmol) in dry THF (8 mL) was treated with NaH (28.8 mg, 1.20 mmol) and monoaldehyde **6** (137 mg, 256 μmol) was added. The crude product was purified by column chromatography (silica gel, PE/DCM=2:1 + 2 % triethylamine, *R*_f_=0.45) to yield the desired compound **Y_NBu_** as an orange viscous oil (90.9 mg, 54.3 μmol, 68 %).^1^H NMR (600 MHz, CDCl_3_) *δ* 7.61–7.37 (m, 5 H), 7.33 (d, *J=*8.4 Hz, 4 H), 7.26–7.03 (m, 11 H), 7.03–6.89 (m, 4 H), 6.82–6.65 (m, 1 H), 6.62–6.15 (m, 7 H), 4.16–3.60 (m, 12 H), 3.18 (m, 12 H), 1.92–1.63 (m, 12 H), 1.48 (m, 24 H), 1.41–1.26 (m, 24 H), 1.25–1.07 (m, 12 H), 0.95–0.73 (m, 36 H). ^13^C NMR (150 MHz, CDCl_3_) *δ* 153.4, 151.4, 150.8, 147.8, 146.5, 138.9, 131.0, 130.3, 129.2, 128.6, 128.4, 128.3, 127.9, 127.7, 125.9, 125.8, 125.3, 124.3, 124.1, 118.4, 115.4, 114.0, 112.6, 112.0, 111.7, 111.2, 110.1, 69.8, 50.9, 31.8, 29.6, 27.8, 26.1, 22.8, 20.5, 16.0, 14.2. IR (cm^−1^): 2953, 2926, 2857, 1607, 1519, 1366, 1185, 1030, 962, 805, 523. HRMS (MALDI): *m*/*z* [*M*+H]^+^ calcd for C_114_H_165_N_3_O_6_ 1673.2777, found 1673.293. Elemental analysis calcd (%) for C_114_H_165_N_3_O_6_: C 81.82; H 9.94; N 2.51; found: C 81.60, H 10.76, N 2.45.

### 1,4‐Bis((*E*)‐2,5‐bis(hexyloxy)‐4‐((*E*)‐2‐(pyridin‐4‐yl)vinyl)styryl)benzene (L_2b_)

Under a nitrogen atmosphere, the bisphosphonate **9** (45.4 mg, 120 μmol) was dissolved in dry THF (10 mL) and the solution was cooled to 0 °C. NaH (28.8 mg, 1.20 mmol) was added carefully and the mixture was stirred at 0 °C for 40 min before monoaldehyde **5** (103 mg, 252 μmol) was added slowly. The reaction mixture was then allowed to warm to RT and further stirred for 2 days. After removing THF on a rotary evaporator, the residues were purified on silica gel column (silica gel, PE/EA=20:1, *R*_f_=0.19) to yield the desired compound **L_2b_** as an orange solid (87.9 mg, 98.9 μmol, 82 %). M.p.: 147–148 °C. ^1^H NMR (600 MHz, CDCl_3_) *δ* 8.53 (m, 4 H), 7.68 (d, *J=*16.4 Hz, 2 H), 7.61–7.42 (m, 6 H), 7.38 (d, *J=*6.0 Hz, 3 H), 7.22–6.97 (m, 9 H), 4.20–3.74 (m, 8 H), 1.99–1.73 (m, 8 H), 1.70–1.48 (m, 8 H), 1.50–1.21 (m, 16 H), 1.09–0.80 (m, 12 H). ^13^C NMR (150 MHz, CDCl_3_) *δ* 151.7, 151.1, 150.2, 149.7, 145.4, 137.3, 129.2, 128.3, 128.2, 127.0, 126.0, 125.5, 124.1, 123.2, 120.9, 111.1, 110.5, 69.6, 31.7, 29.6, 29.5, 26.1, 22.8, 14.2. IR (cm^−1^): 2931, 2853, 1590, 1397, 1207, 1034, 961, 847, 726, 525. HRMS (MALDI): *m*/*z* [M]^+^ calcd for C_60_H_76_N_2_O_4_ 888.5805, found 888.5835.

### 4‐((*E*)‐4‐((*E*)‐3,5‐Bis((*E*)‐2,5‐bis(hexyloxy)‐4‐((*E*)‐styryl)styryl)styryl)‐2,5‐bis(hexyloxy)styryl)pyridine (Y_1_)

Under a nitrogen atmosphere, the triphosphonate **7** (42.3 mg, 80 μmol) was dissolved in dry THF (10 mL) and the solution was cooled to 0 °C. NaH (28.8 mg, 1.2 mmol) was added carefully and the mixture was stirred at 0 °C for 40 min before monoaldehyde **5** (35.4 mg, 86.8 μmol) was added slowly. The reaction mixture was stirred overnight at room temperature. After removing THF in vacuo, the residues containing **10** were dried and used in the next step without further purification. To a solution of **10** in dry THF (8 mL) was added NaH (28.8 mg, 1.20 mmol) at 0 °C. The mixture was stirred at 0 °C for 40 min, and then another monoaldehyde **4** (71.9 mg, 176 μmol) was added. The reaction mixture was then allowed to warm to RT and further stirred for 2 days. After removing THF on a rotary evaporator, the crude product was purified by column chromatography (silica gel, PE/EA=1:1, *R*_f_=0.49) to yield the desired compound **Y_1_** as an orange solid (29.8 mg, 23.1 μmol, 28 % over two steps). M.p.: 71–72 °C. ^1^H NMR (600 MHz, CDCl_3_) *δ* 8.69 (d, *J=*5.8 Hz, 2 H), 7.82 (d, *J=*16.4 Hz, 1 H), 7.74–7.60 (m, 12 H), 7.49 (m, 6 H), 7.37 (dd, *J=*16.3, 7.8 Hz, 4 H), 7.34–7.23 (m, 9 H), 7.19 (d, *J=*16.4 Hz, 1 H), 4.20 (dt, *J=*10.9, 6.5 Hz, 12 H), 2.07–1.98 (m, 12 H), 1.74–1.66 (m, 12 H), 1.57–1.47 (m, 24 H), 1.08–0.99 (m, 18 H). ^13^C NMR (150 MHz, CDCl_3_) *δ* 151.8, 151.2, 150.1, 145.5, 138.8, 138.5, 138.0, 129.8, 128.9, 128.7, 128.4, 128.31, 127.5, 127.0, 126.6, 125.9, 125.5, 124.3, 123.6, 120.9, 111.0, 110.7, 69.7, 31.7, 29.5, 26.1, 22.8, 14.2. IR (cm^−1^): 2925, 2856, 2359, 1590, 1420, 1202, 1029, 960, 690, 507. HRMS (MALDI): *m*/*z* [*M*+H]^+^ calcd for C_89_H_114_NO_6_ 1292.8646, found 1292.963.

### 4,4′‐({5‐[(*E*)‐2‐{2,5‐Bis(hexyloxy)‐4‐[(*E*)‐2‐phenylethenyl]phenyl}ethenyl]‐1,3‐phenylene}bis{[(*E*)ethene‐2,1‐diyl][2,5‐bis(hexyloxy)‐4,1‐phenylene](*E*)ethene‐2,1‐diyl})dipyridine (Y_2_)

Under a nitrogen atmosphere, the triphosphonate **7** (42.3 mg, 80.0 μmol) was dissolved in dry THF (8 mL) and the solution was cooled to 0 °C. NaH (28.8 mg, 1.20 mmol) was added carefully and the mixture was stirred at 0 °C for 40 min before monoaldehyde **5** (70.8 mg, 174 μmol) was added slowly. The reaction mixture was stirred overnight at room temperature. After removing THF on a rotary evaporator, the residues containing **11** were dried and used in the next step without further purification. To a solution of 11 in dry THF (8 mL) was added NaH (28.8 mg, 1.20 mmol) at 0 °C. The mixture was stirred at 0 °C for 40 min, and then another monoaldehyde **4** (36.1 mg, 88.0 μmol) was added. The reaction mixture was then allowed to warm to RT and further stirred for 2 days. After removing THF on a rotary evaporator, the crude product was purified by column chromatography (silica gel, EE/MeOH=50:1, *R*_f_=0.58) to yield the desired compound **Y_2_** as an orange solid (46.4 mg, 35.8 μmol, 44 % over two steps). M.p.: 121–122 °C. ^1^H NMR (600 MHz, CDCl_3_) *δ* 8.70 (d, *J=*5.8 Hz, 4 H), 7.81 (d, *J=*16.4 Hz, 2 H), 7.75–7.58 (m, 9 H), 7.56–7.45 (m, 6 H), 7.42–7.32 (m, 4 H), 7.27 (m, 7 H), 7.19 (d, *J=*16.4 Hz, 2 H), 4.21 (m, 12 H), 2.10–1.90 (m, 12 H), 1.82–1.62 (m, 12 H), 1.62–1.42 (m, 24 H), 1.04 (m, 18 H). ^13^C NMR (150 MHz, CDCl_3_) *δ* 151.7, 151.3, 151.2, 150.2, 145.4, 138.8, 138.6, 138.0, 129.7, 128.9, 128.8, 128.3, 128.2, 127.6, 127.2, 126.9, 126.6, 126.1, 125.6, 124.3, 124.1, 123.6, 120.9, 111.1, 110.8, 69.7, 31.7, 29.6, 26.1, 22.8, 14.2. IR (cm^−1^): 2926, 2856, 2360, 1589, 1419, 1203, 962, 690, 527. HRMS (MALDI): *m*/*z* [*M*+H]^+^ calcd for C_88_H_113_N_2_O_6_ 1293.8599, found 1293.919.

CCDC  1959608 (**L_2b_**), 1959609 (**Y_0_**), and 1959610 (**Y_3_**) contain the supplementary crystallographic data for this paper. These data are provided free of charge by The Cambridge Crystallographic Data Centre.

## Conflict of interest

The authors declare no conflict of interest.

## Supporting information

As a service to our authors and readers, this journal provides supporting information supplied by the authors. Such materials are peer reviewed and may be re‐organized for online delivery, but are not copy‐edited or typeset. Technical support issues arising from supporting information (other than missing files) should be addressed to the authors.

Supplementary
