# Supplementary material for: Linear and Star‐Shaped Extended Di‐ and Tristyrylbenzenes: Synthesis, Characterization and Optical Response to Acid and Metal Ions
Source: Chemistry. 2020 Jun 8;26(36):8137–43. doi: 10.1002/chem.202000893 (PMC7383513; doi:10.1002/chem.202000893)
Supplement: Supplementary file 1 — Supplementary [file CHEM-26-8137-s001.pdf]

Chemistry–A European Journal

Supporting Information

Linear and Star-Shaped Extended Di- and Tristyrylbenzenes: Synthesis, Characterization and Optical Response to Acid and Metal Ions

Hao Zhang, Eugen A. Kotlear, Soh Kushida, Steffen Maier, Frank Rominger, Jan Freudenberg,* and Uwe H. F. Bunz*[a]

Contents

S1. Materials and methods 2
S2. Synthetic details and analytical data for intermediates 3
S3. Crystal data and properties 4
S4. Formation of liquid crystalline 8
S4. DFT calculations 9
S5. Photophsical data of SBs in selected solvents 11
S6. Absorption and emission spectra for the TFA titrations 13
S7. NMR-spectroscopy 14
S8. References 26

S1. Materials and methods

All reagents were purchased from Sigma-Aldrich, Merck, ABCR or Fisher Scientific and used without further purification. Oxygen or moisture sensitive reactions were carried out in oven-dried glassware under nitrogen atmosphere using a standard Schlenk technique. Absolute solvents were dried by a MB SPS-800 solvent purification system using drying columns. Thin layer chromatography (TLC) was performed on Polygram SIL G/UV254 plates from Macherey, Nagel GmbH &Co. KG, Düren (Germany) and examined under ultraviolet light irradiation (254 and 365 nm). Column chromatography was carried out using silica gel from Sigma-Aldrich (particle size: 0.063-0.200 mm) or from Macherey-Nagel GmbH & Co. KG, Düren (Germany) (particle size: 0.040-0.063 mm). NMR spectra (^{1}H,^{13}C) were recorded at 298 K on Bruker Avance III 300, Bruker Avance III 400 or Bruker Avance III 600 spectrometer. Chemical shifts (δ) are reported in parts per million (ppm) relative to solvent residual peak. IR spectra were recorded neat on Jasco FT/IR-4100 spectrometer and reported in wavenumbers (cm^{-1}). Mass spectra (MS) were obtained by direct analysis in real time (DART), matrix-assisted laser desorption/ionization or electron impact (EI) experiments on a Bruker ApexQe hybrid 9.4 TFT-ICR-MS at the Mass Spectrometry Facility of the University of Heidelberg. Crystal structure analysis was performed on a Bruker APEX-II Quazar diffractometer. Absorption spectra were recorded using a Jasco UV/VIS V-660 spectrophotometer and emission spectra were measured on a Jasco FP-6500 spectrofluorometer. Quantum yields were measured on a PTI Quantamaster 40. Quantum lifetimes were determined on a Horiba FluoroCube-01-NLlifetime spectrofluorometer with emission monochromator (Seya-Namiokatype, 200 nm to 800 nm) and diode excitation (Nano-LED N-375L, 375 ± 10 nm, < 200 ps). Melting points were determined in glass capillaries with a Melting Point Apparatus MEL-TEMP (Electrothermal, Rochford, UK) and were reported uncorrected. Photographs were taken under normal light and UV-light irradiation (λ = 365 nm) using a Canon EOS 7D digital camera. Theory calculations were carried out using a B3LYP/6-311++G**basis set on Gaussian16. The star-shaped compounds were observed in the heating and cooling processes using an LV100i Pol polarizing optical microscope (POM) equipped with a digital Nikon camera and a temperature-controllable Linkam hot stage.

S2. Synthetic details and analytical data for intermediates

1,4-Dibromo-2,5-bis(hexyloxy)benzene (2). An oven dried 250 mL Schlenk flask equipped with a stir-bar was cooled under N_2, charged with **1** (8.20 g, 30.5 mmol) and K_2CO_3 (21.1 g, 153 mmol), and backfilled with nitrogen. A N_2 degassed solution of 1-bromohexane (11.6 g, 70.2 mmol) in DMF (60 mL) was then added and the reaction mixture was heated to 110 °C for 48 h. The reaction mixture was then poured into 300 mL of H_2O and the white solid **2** was collected (12.2 g, 27.9 mmol, 92%). ^{1}H NMR (300 MHz, $CDCl_3$) δ 7.09 (s, 2H), 3.95 (t, *J* = 6.5 Hz, 4H), 1.93 – 1.69 (m, 4H), 1.64 – 1.42 (m, 4H), 1.34 (m, 8H), 0.91 (t, *J* = 7.0 Hz, 6H). Consistent with reported values.[1]

4-Bromo-2,5-bis(hexyloxy)benzaldehyde (3).[2] 2,5-Dihexyloxy1,4-dibromobenzene **2** (8.70 g, 20.0 mmol) was loaded into a 500 mL Schlenk flask and vacuum purged with argon three times. Anhydrous tetrahydrofuran (200 mL) was added and the flask was placed into a bath of dry ice and 2-propanol. *n*-Butyllithium (10.0 mL) in hexane was added dropwise and the reaction was stirred for 30 minutes. Anhydrous dimethylformamide (4.5 mL) was then added and the reaction was allowed to warm to room temperature for 4 hours. The reaction was quenched carefully with water and extracted with diethylether for three times. Column chromatography (silica gel, diethylether: petroleum ether = 1:20, R_f = 0.40) afforded **3** as a colorless solid (3.80 g, 9.80 mmol, 49%). ^{1}H NMR (300 MHz, $CDCl_3$) δ 10.41 (s, 1H), 7.31 (s, 1H), 7.22 (s, 1H), 4.02 (m, 4H), 1.95 – 1.69 (m, 4H), 1.64 – 1.42 (m, 4H), 1.42 – 1.23 (m, 8H), 0.91 (q, J = 6.2 Hz, 6H).

***N,N*-Dibutyl-4-vinylaniline.**[3] An oven-dried 50 mL Schlenk flask was charged with methyltriphenylphosphonium bromide (2.60 g, 7.92 mmol) and 10 mL dry THF and stirred under N_2. And the flask was placed into a bath of dry ice and 2-propanol. A 2.5 M solution of *n*-butyllithium in hexanes was added via syringe (3.0 mL, 7.10 mmol). After stirring for half an hour, 4-*N, N*-dibutylaminobenzaldehyde (1.00 g, 4.40 mmol) was added. After stirring one day, the reaction mixture was poured into 200 mL of hexanes. The suspension was filtered through celite and the filtrate was concentrated under reduced pressure until it reached a volume of 20 mL. The solution was filtered through an ultrafilter and the hexane was removed completely. A yellow oil was collected (1.01 g, 4.30 mmol, 98%). ^{1}H NMR ($CDCl_3$, 300 MHz) δ 7.22 (d, *J* = 10 Hz, 2 H), 6.55 (m, 3 H), 5.44 (d, *J* = 18 Hz, 1 H), 4.92 (d, *J* = 11.1 Hz, 1 H), 3.22 (t, *J* = 7.6 Hz, 4 H), 1.52 (m, 4 H), 1.32 (m, 4 H), 0.99 (t, *J* = 7.1 Hz, 6H).

2,5-Bis(hexyloxy)-4-styrylbenzaldehyde (4).[4] According to **GP1** a solution of monoaldehyde **3** (694 mg, 1.80 mmol), styrene (214 mg, 2.00 mmol), $Pd(OAc)_2$ (20.2 mg, 90 μmol), tris(*o*-tolyl)phosphine (54.8 mg, 180 μmol) and triethylamine (1.40 mL) in DMF (20 mL) was stirred at 110 °C for 48 h. Column chromatography (silica gel, PE/EA = 20:1, R_f = 0.50) afforded **4** as a yellow solid (111 mg, 272 μmol, 30%). ^{1}H NMR (300 MHz, $CDCl_3$) δ 10.45 (s, 1H), 7.61 – 7.52 (m, 2H), 7.38 (m, 2H), 7.35 – 7.20 (m, 4H), 7.18 (s, 1H), 4.12 (t, *J* = 6.4 Hz, 2H), 4.03 (t, *J* = 6.5 Hz, 2H), 1.93 – 1.78 (m, 4H), 1.53 (m, 4H), 1.45 – 1.30 (m, 8H), 0.98 – 0.86 (m, 6H).

Hexaethyl (benzene-1,3,5-triyltris(methylene))tris(phosphonate) (7).[5] The title compound was synthesized by a previously reported method, and NMR data of the product matched those reported in the literature. ^{1}H NMR (300 MHz, $CDCl_3$) δ 7.00 (s, 3H), 3.89 (m, 12H), 2.98 (d, J = 21.9 Hz, 6H), 1.20 – 1.06 (m, 18H).

Tetraethyl (1,4-phenylenebis(methylene))bis(phosphonate) (9).[6] The title compound was synthesized by a previously reported method, and NMR data of the product matched those reported in the literature. ^{1}H NMR (300 MHz, $CDCl_3$) δ 7.24 (s, 4H), 4.02 (t, *J* = 7.5 Hz, 8H), 3.12 (d, *J* = 20.2 Hz, 4H), 1.23 (t, *J* = 7.2 Hz, 12H).

S3. Crystal data and properties

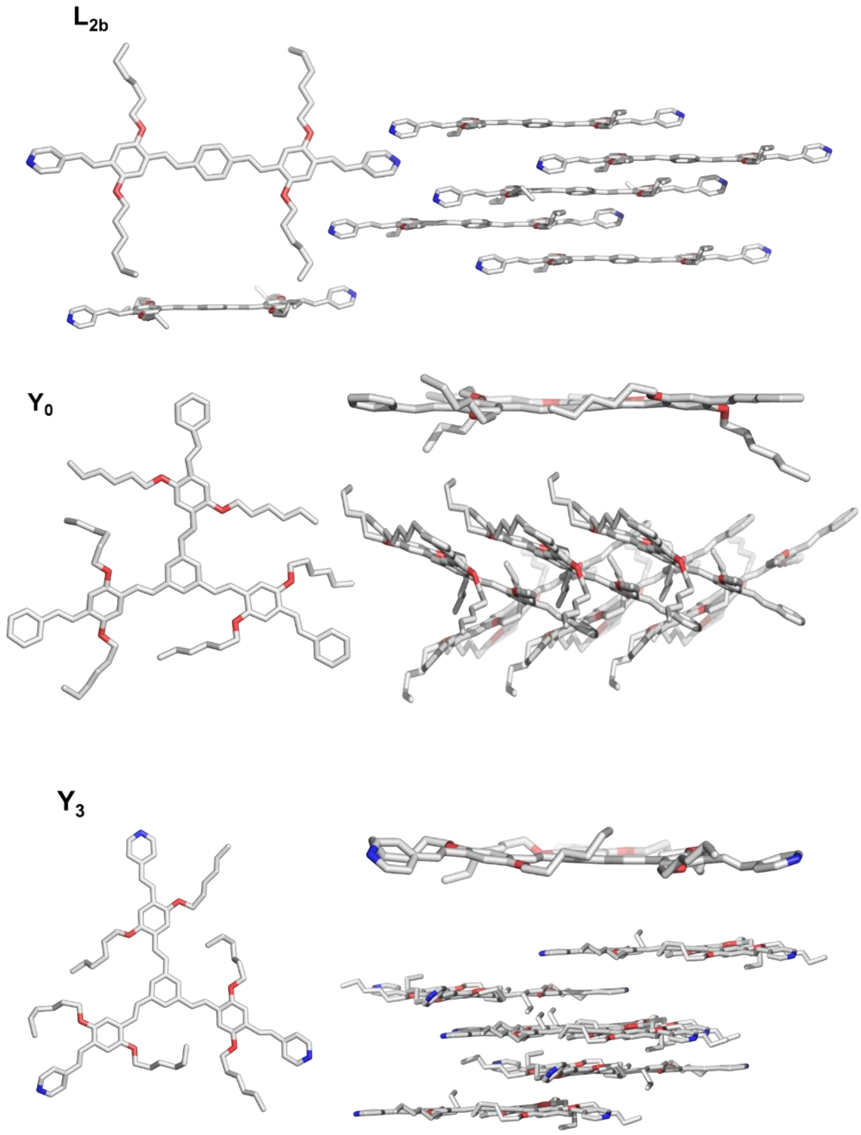

Figure S1. Solid-state structures of $\mathbf{L_{2b}}$, $\mathbf{Y_0}$ and $\mathbf{Y_3}$; side view; visualization of packing.

Table S1. Crystal data and structure refinement for **L_{2b}**.

CCDC	1959608
Empirical formula	$C_{60}H_{76}N_2O_4$
Formula weight	889.22
Temperature	110(2) K
Wavelength	1.54178 Å
Crystal system	triclinic
Space group	$P\bar{1}$
Z	2
Unit cell dimensions	a = 12.1325(9) Å α =99.905(6) deg.
	b = 15.0247(11) Å β = 107.287(6) deg.
	c = 15.1022(11) Å γ =95.066(6) deg.
Volume	2560.8(3) Å^3
Density (calculated)	1.15 g/cm^3
Absorption coefficient	0.55 mm^{-1}
Crystal shape	brick
Crystal size	0.155 x 0.137 x 0.066 mm^3
Crystal colour	orange
Theta range for data collection	3.1 to 66.9 deg.
Index ranges	$-11\leq h\leq 14$, $-17\leq k\leq 15$, $-16\leq l\leq 17$
Reflections collected	19962
Independent reflections	8562 (R(int) = 0.0300)
Observed reflections	4917 ($I > 2\sigma(I)$)
Absorption correction	Semi-empirical from equivalents
Max. and min. transmission	1.72 and 0.56
Refinement method	Full-matrix least-squares on F^2
Data/restraints/parameters	8562 / 1478 / 715
Goodness-of-fit on F^2	1.05
Final R indices (I>2sigma(I))	R1 = 0.077, wR2 = 0.213
Largest diff. peak and hole	0.58 and -0.36 eÅ^{-3}

Table S2. Crystal data and structure refinement for **Y_0**.

CCDC	1959609
Empirical formula	$C_{90}H_{114}O_6$
Formula weight	1291.81
Temperature	100(2) K
Wavelength	1.54178 Å
Crystal system	monoclinic
Space group	$P2_1/c$
Z	4
Unit cell dimensions	a = 15.8983(5) Å α = 90 deg.
	b =9.8893(3) Å β =92.562(3) deg.
	c = 49.2710(16) Å γ = 90 deg.
Volume	7738.8(4) $Å^3$
Density (calculated)	1.11 g/cm^3
Absorption coefficient	0.52 mm^{-1}
Crystal shape	plate
Crystal size	0.220 x 0.088 x 0.050 mm^3
Crystal colour	neonyellow
Theta range for data collection	4.6 to 51.1 deg.
Index ranges	$-16\leq h\leq 12$, $-9\leq k\leq 9$, $-49\leq l\leq 46$
Reflections collected	25405
Independent reflections	8039 (R(int) = 0.0497)
Observed reflections	5304 (I > 2σ(I))
Absorption correction	Semi-empirical from equivalents
Max. and min. transmission	1.58 and 0.51
Refinement method	Full-matrix least-squares on F^2
Data/restraints/parameters	8039 / 1099 / 940
Goodness-of-fit on F^2	1.03
Final R indices (I>2sigma(I))	R1 = 0.101, wR2 = 0.271
Largest diff. peak and hole	0.48 and -0.26 $eÅ^{-3}$

Table S3. Crystal data and structure refinement for **Y_3**.

CCDC	1959610	
Empirical formula	$C_{87}H_{111}N_3O_6$	
Formula weight	1294.78	
Temperature	110(2) K	
Wavelength	1.54178 Å	
Crystal system	triclinic	
Space group	$P\bar{1}$	
Z	2	
Unit cell dimensions	a = 14.1922(17) Å	α =94.03(1) deg.
	b = 14.7488(17) Å	β =93.06(1) deg.
	c = 19.855(3) Å	γ =113.568(9) deg.
Volume	3784.9(8) $Å^3$	
Density (calculated)	1.14 g/cm^3	
Absorption coefficient	0.54 mm^{-1}	
Crystal shape	brick	
Crystal size	0.260 x 0.157 x 0.075 mm^3	
Crystal colour	yellow	
Theta range for data collection	3.4 to 49.1 deg.	
Index ranges	$-13 \le h \le 10$, $-12 \le k \le 14$, $-19 \le l \le 19$	
Reflections collected	21659	
Independent reflections	7396 (R(int) = 0.0325)	
Observed reflections	4270 ($I > 2\sigma(I)$)	
Absorption correction	Semi-empirical from equivalents	
Max. and min. transmission	1.65 and 0.57	
Refinement method	Full-matrix least-squares on F^2	
Data/restraints/parameters	7396 / 1065 / 865	
Goodness-of-fit on F^2	2.38	
Final R indices (I>2sigma(I))	R1 = 0.154, wR2 = 0.371	
Largest diff. peak and hole	0.63 and -0.31 $eÅ^{-3}$	

S4. Formation of liquid crystalline

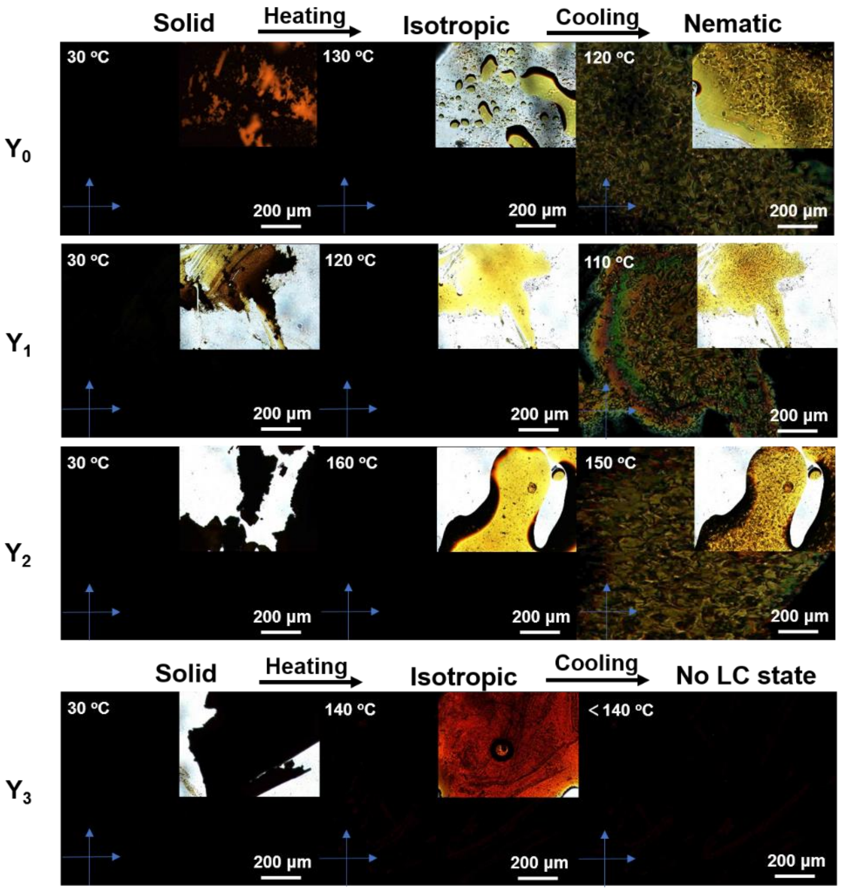

Figure S2. Polarization micrographs under crossed polarizers at given temperatures while heating and cooling samples of **Y_{0-3}**. Insets: Optical micrographs from the corresponding samples.

S4. TDDFT/DFT calculations

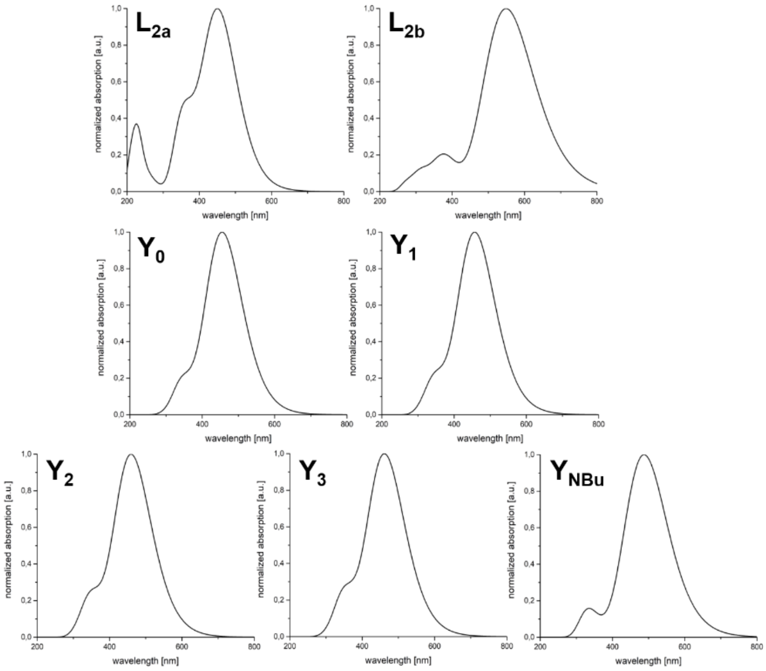

Compounds	λ_{abs} [nm][a]	calcd λ_{abs} [nm]
**L_{2a}**	329 (2.37), 400 (2.88)	450
**L_{2b}**	432 (7.31)	550
**Y_0**	329 (6.17), 401 (11.0)	454
**Y_1**	330 (5.68), 403 (9.89)	458
**Y_2**	331 (7.13), 405 (12.4)	460
**Y_3**	348 (2.29), 401 (2.86)	460
**Y_{NBu}**	326 (1.91), 427 (8.64)	488

[a] measured in THF.

Figure S3. UV-Vis spectra calculated from the optimized geometries of the ground states using TDDFT B3LYP/def2svp with a list of their measured and calculated maxima.

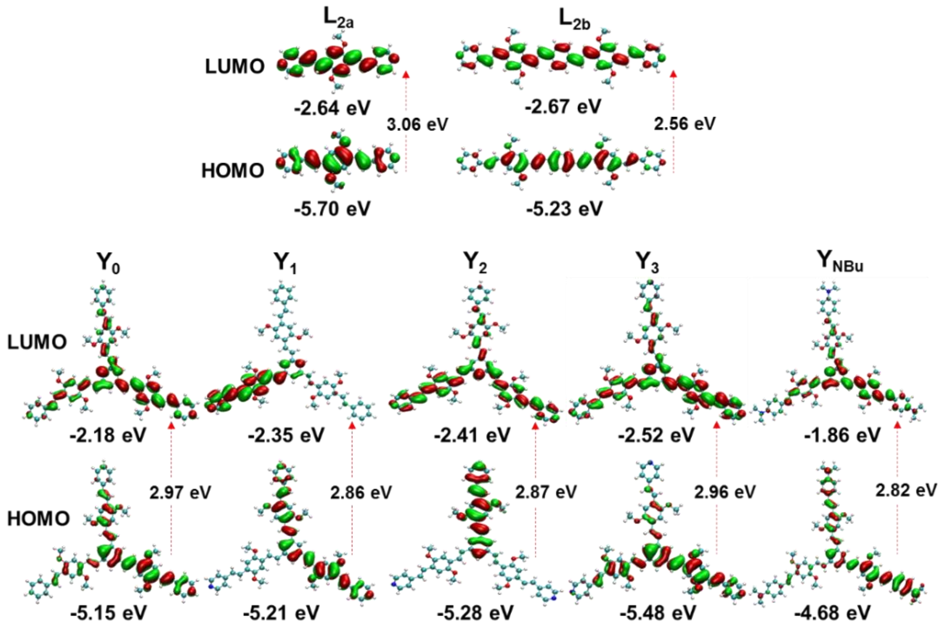

Figure S4. Frontier molecular orbital levels of SBs (DFT, B3LYP/6-311++G**). Aliphatic chain approximated by a methyl group.

S5. Photophsical data of SBs in selected solvents

$$\Delta\nu = \Delta\nu_{abs} - \Delta\nu_{em} = \frac{2(\mu_e-\mu_g)^2\Delta f}{hca^3} + \text{constant} \qquad (1)$$

$$\Delta f = \frac{\varepsilon-1}{2\varepsilon+1} - \frac{n^2-1}{2n^2+1} \qquad (2)$$

In eq 1, $\Delta\nu$ is the Stokes shift; $\Delta\nu_{abs}$ and $\Delta\nu_{em}$ represent the wavenumbers of absorption and emission peaks; h is Planck's constant; c is the speed of light and a corresponds to the Onsager cavity radius; μ_e and μ_g are the excited and ground state dipole moments. In eq 2, Δf is the orientational polarizability of solvent; ε is the dielectric constant; n is refractive index of the solvent. Table S4 shows the photophsical data of SBs in selected solvents. The relationship of the Stokes shift ($\Delta\nu$) and the orientational polarizability (Δf) of the solvents was plotted in Figure S5, and the corresponding slopes of Lippert-Mataga plot for SBs were summarized in Table S5.

Table S4. Photophsical data of SBs in selected solvents.

	Hexane			Toluene			DCM			Acetonitrile			DMSO		
	λ_{ab} (nm)	λ_{em} (nm)	$\Delta\upsilon_{st}$ (cm^{-1})	λ_{ab} (nm)	λ_{em} (nm)	$\Delta\upsilon_{st}$ (cm^{-1})	λ_{ab} (nm)	λ_{em} (nm)	$\Delta\upsilon_{st}$ (cm^{-1})	λ_{ab} (nm)	λ_{em} (nm)	$\Delta\upsilon_{st}$ (cm^{-1})	λ_{ab} (nm)	λ_{em} (nm)	$\Delta\upsilon_{st}$ (cm^{-1})
**L_{2a}**	393	442	2821	398	459	3339	402	466	3416	398	471	3894	411	476	3322
**L_{2b}**	424	475	2532	432	489	2698	434	499	3001	428	516	3985	444	532	3726
**Y_0**	396	440	2525	402	449	2604	403	454	2787	401	452	2814	407	459	2784
**Y_1**	397	443	2616	403	454	2787	405	463	3093	402	465	3370	409	465	2945
**Y_2**	400	445	2528	405	457	2810	407	466	3111	405	467	3278	412	473	3130
**Y_3**	398	444	2603	404	455	2774	406	461	2939	405	458	2857	408	463	2912
**Y_{NBu}**	415	465	2591	424	483	2881	428	536	4708	426	560	5617	436	566	5268

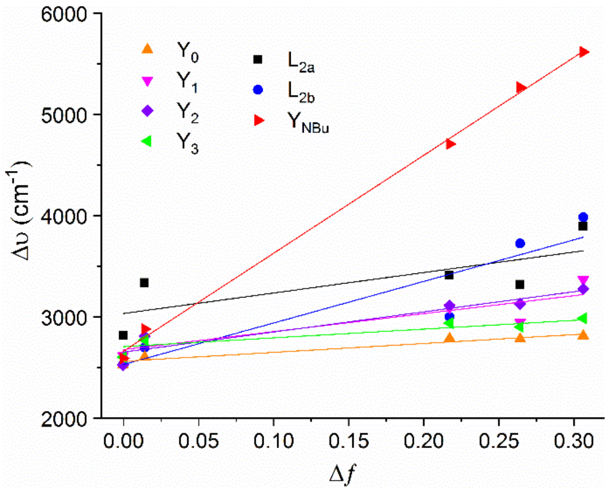

Figure S5. Stokes shifts ($\Delta\upsilon$) of as a function of solvent polarity parameter (Δf).

Table S5. Slopes of Lippert-Mataga plot for SBs.

Compounds	Slope (cm^{-1})
**L_{2a}**	2022
**L_{2b}**	4104
**Y_0**	878
**Y_1**	1774
**Y_2**	1987
**Y_3**	892
**Y_{NBu}**	9673

S6. Absorption and emission spectra for the TFA titrations

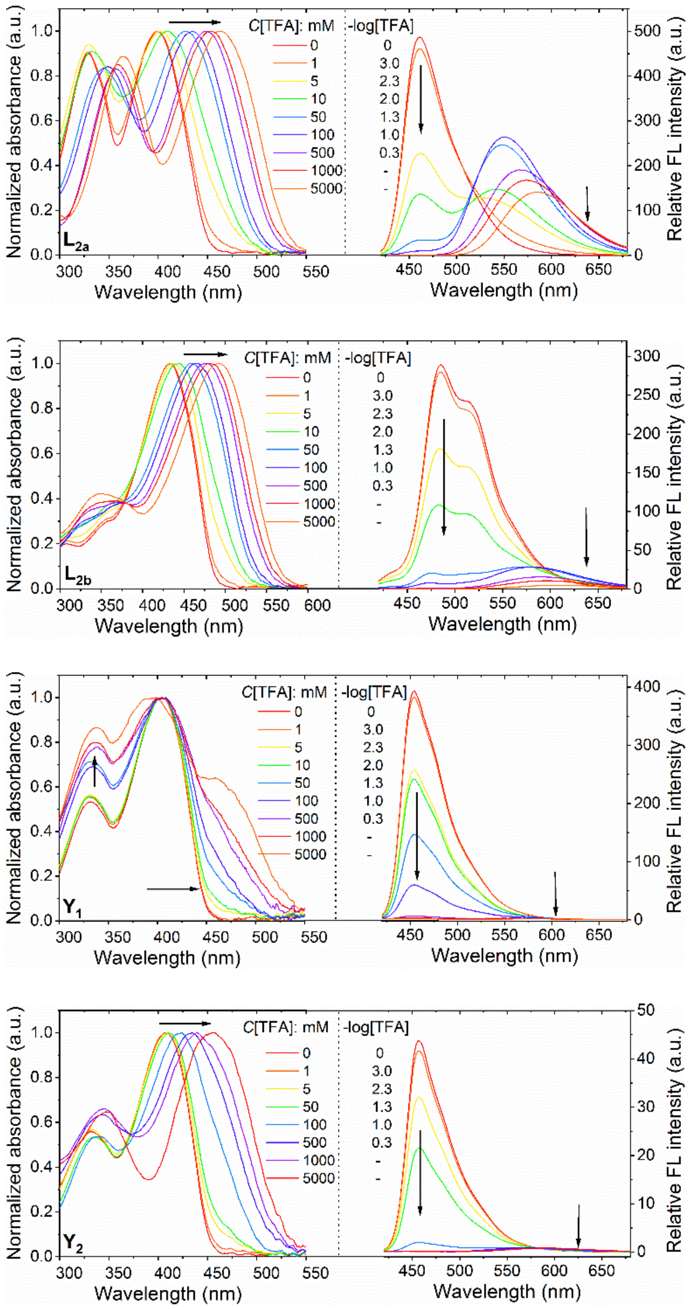

Figure S6. Normalized UV/vis absorption (left) and emission (right) spectra for the titrations of $\mathbf{L_{2a}}$, $\mathbf{L_{2b}}$, $\mathbf{Y_1}$ and $\mathbf{Y_2}$ in THF with different concentration of TFA.

S7. NMR-spectroscopy

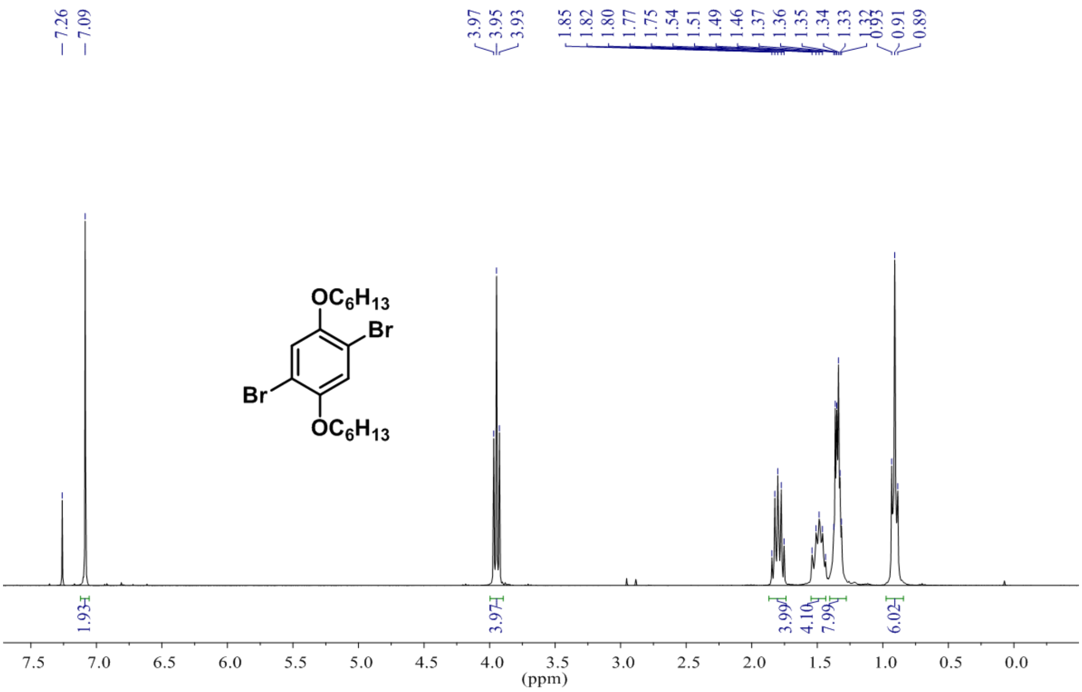

Figure S7. ^{1}H NMR spectra of **2** in $CDCl_3$.

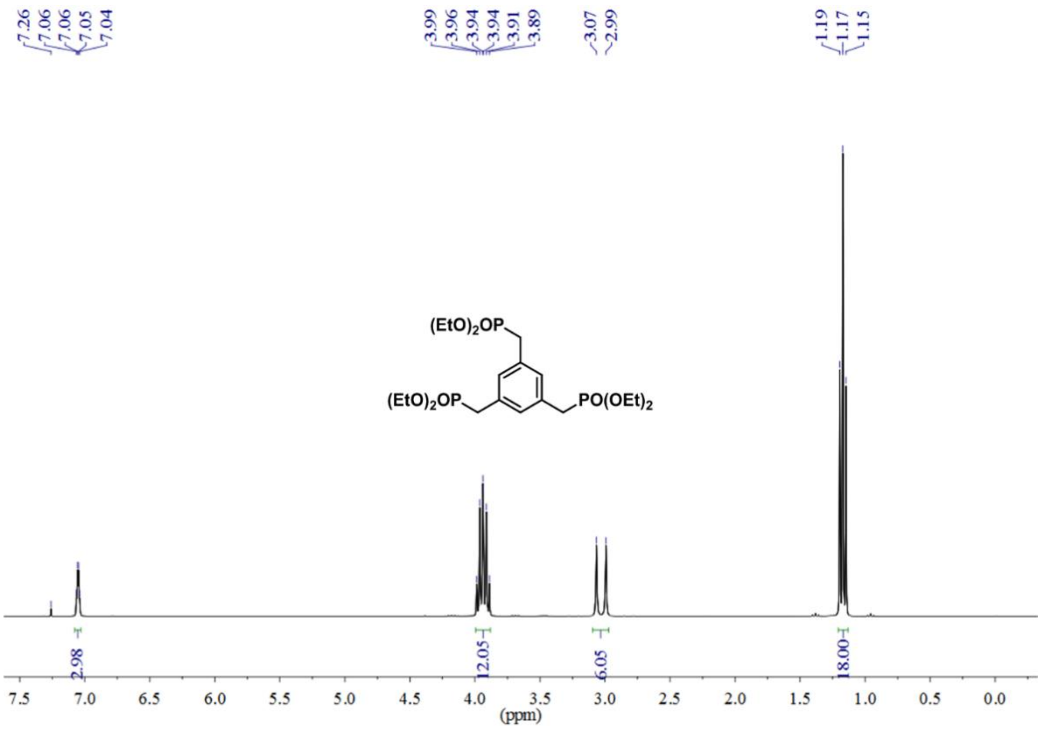

Figure S8. ^{1}H NMR spectra of **10** in $CDCl_3$.

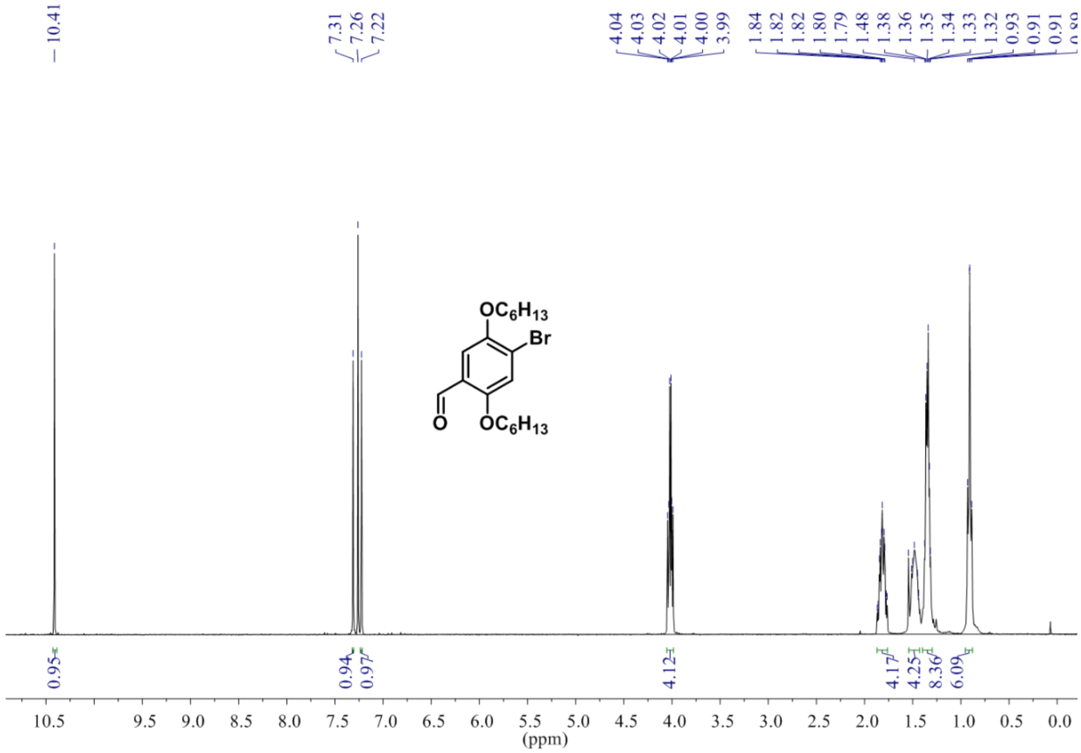

Figure S9. ^{1}H NMR spectra of **3** in $CDCl_3$.

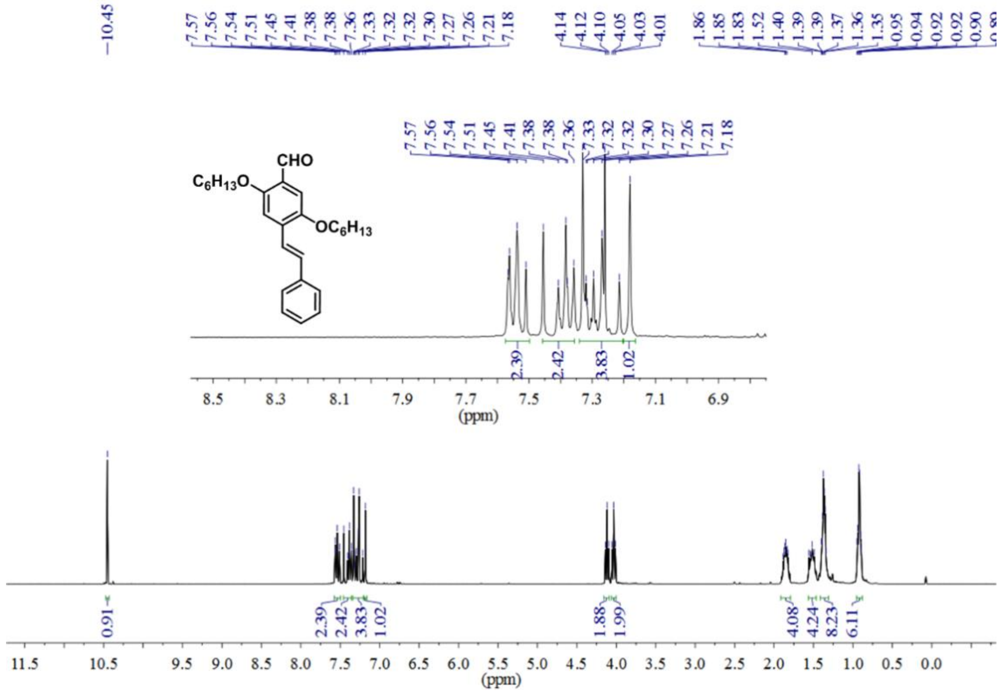

Figure S10. ^{1}H NMR spectra of **4** in $CDCl_3$.

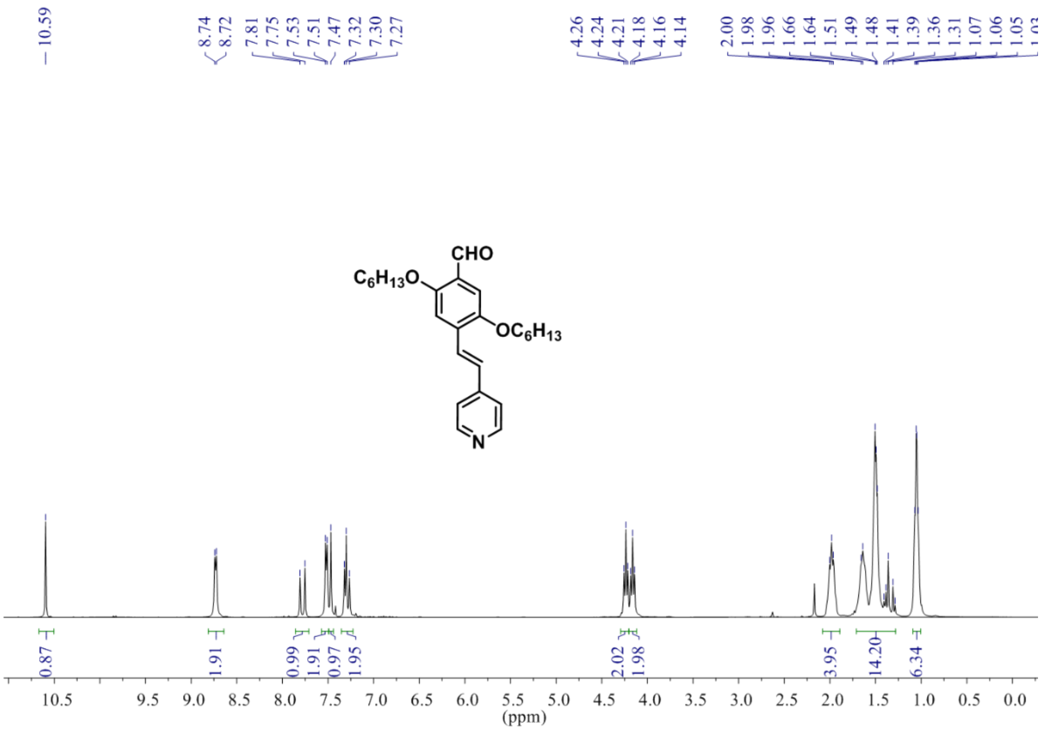

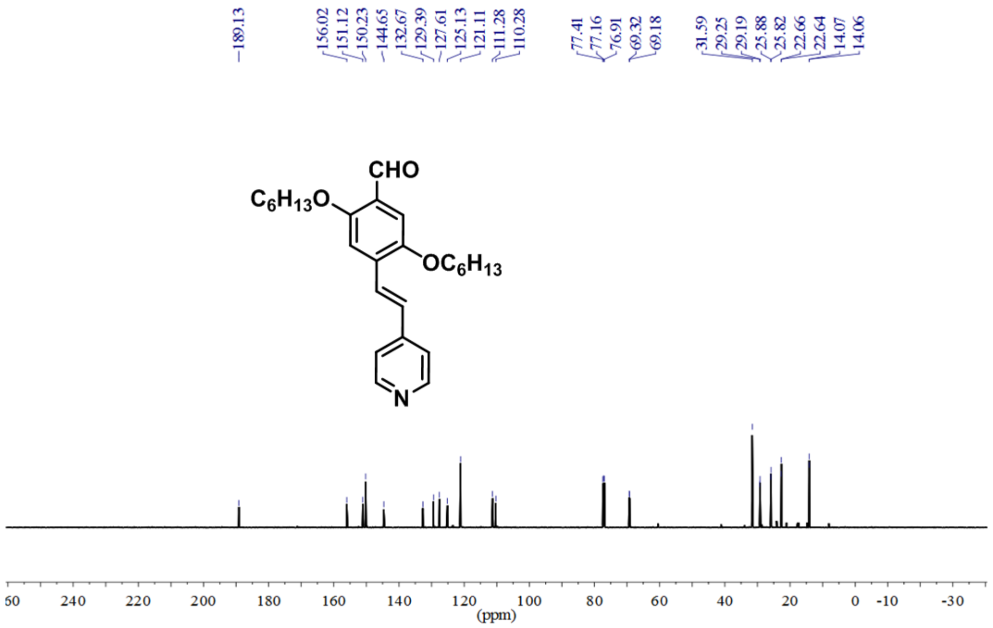

Figure S11. ^{1}H NMR (top) and ^{13}C NMR (bottom) spectra of **5** in $CDCl_3$.

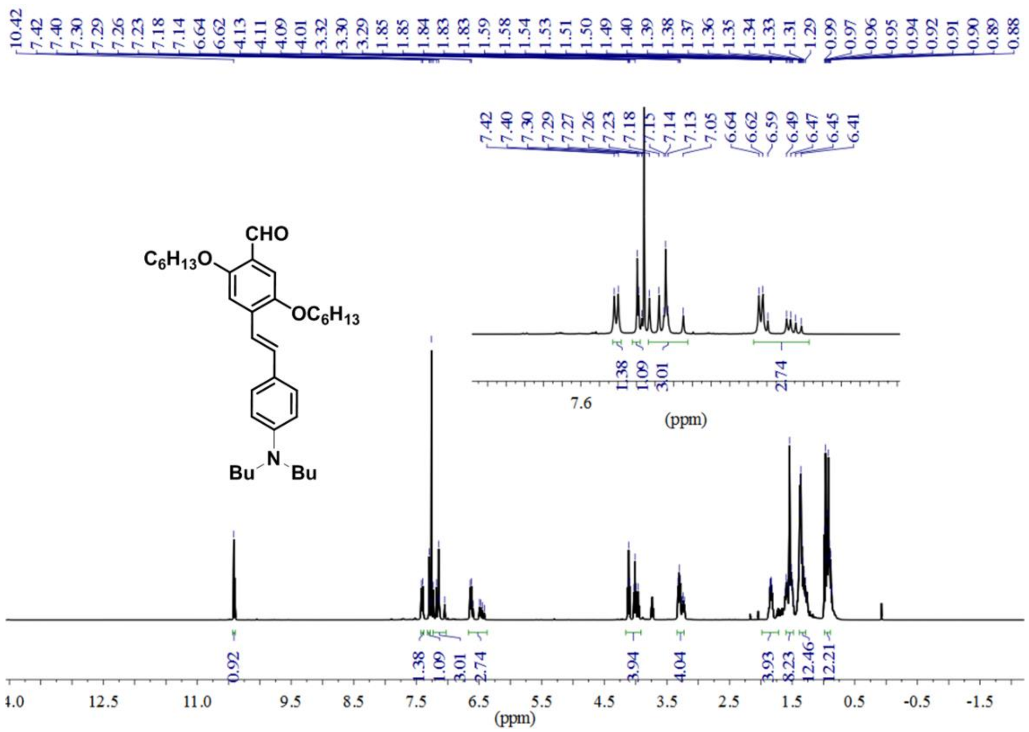

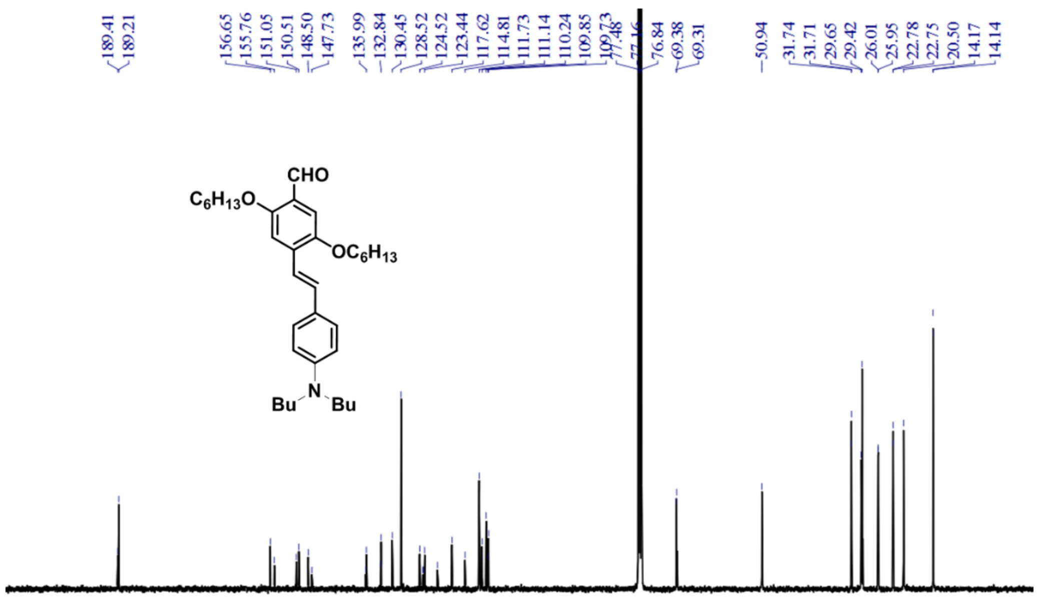

Figure S12. ^{1}H NMR (top) and ^{13}C NMR (bottom) spectra of **6** in $CDCl_3$.

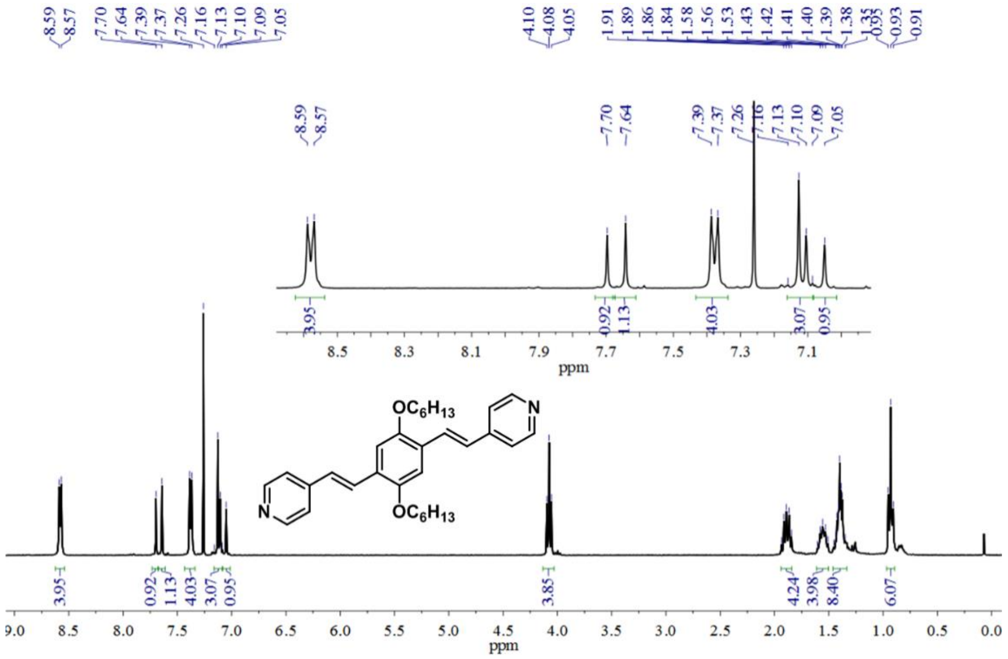

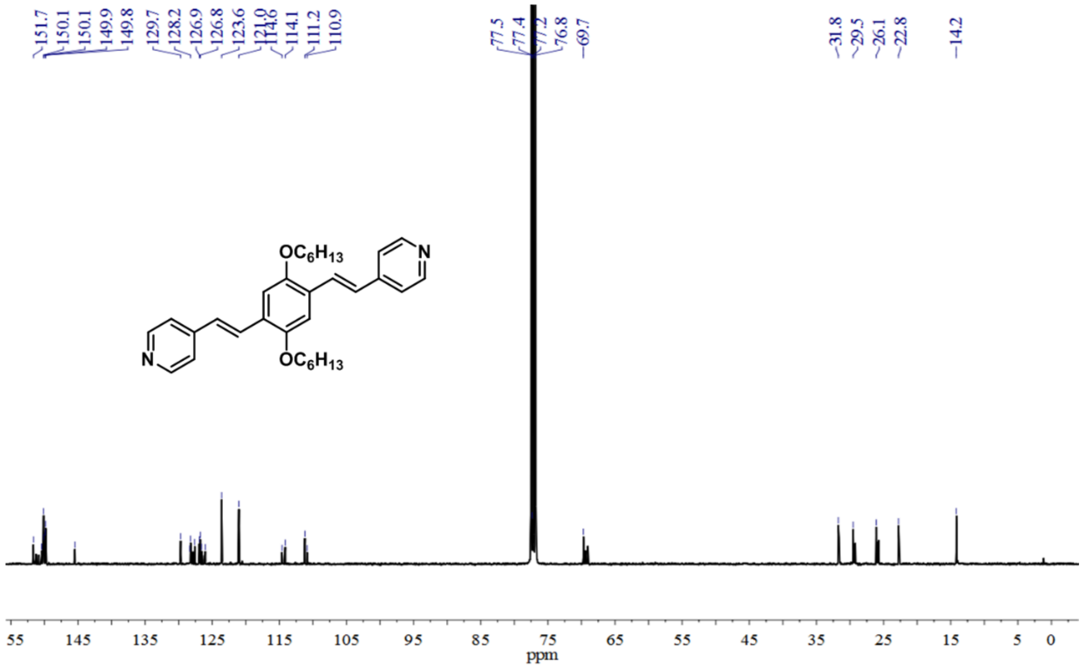

Figure S13. ^{1}H NMR (top) and ^{13}C NMR (bottom) spectra of $\mathbf{L_{2a}}$ in $CDCl_3$.

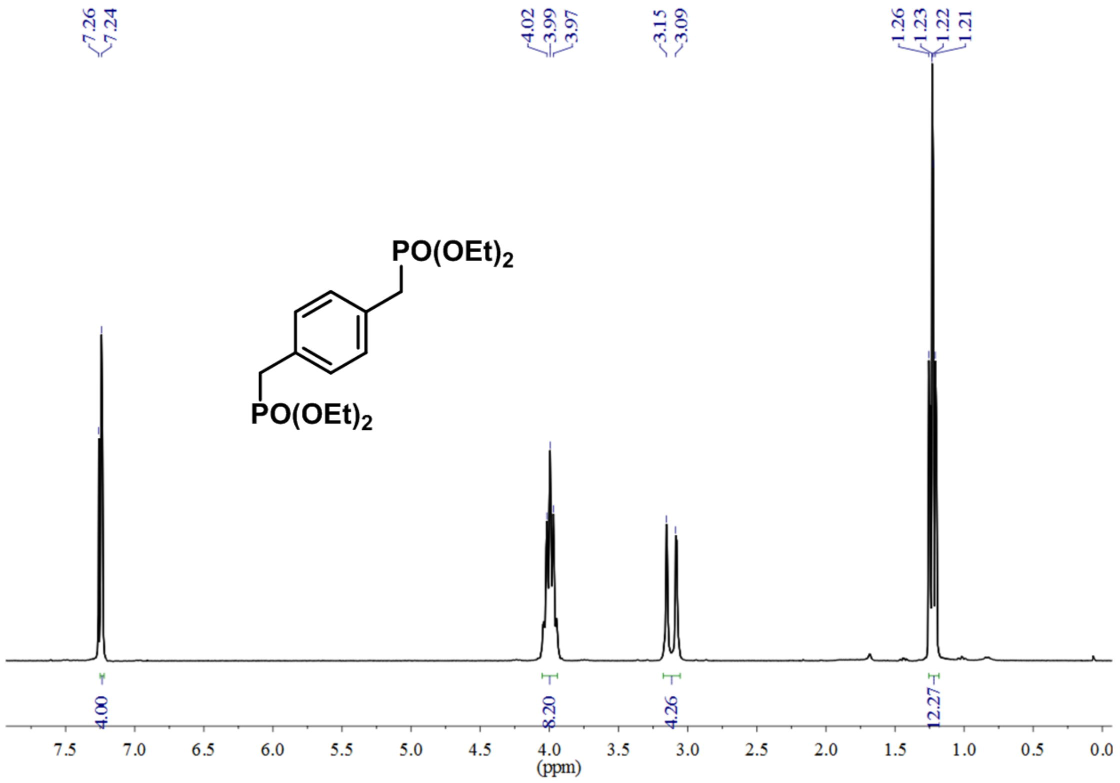

Figure S14. ^{1}H NMR spectra of **9** in $CDCl_3$.

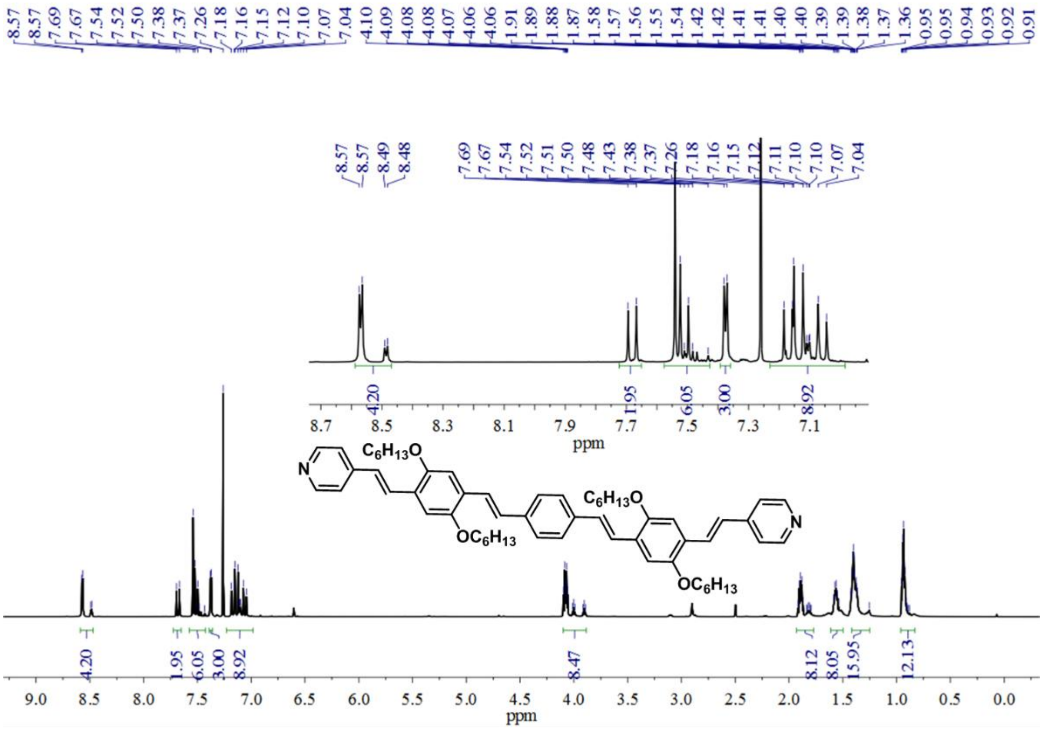

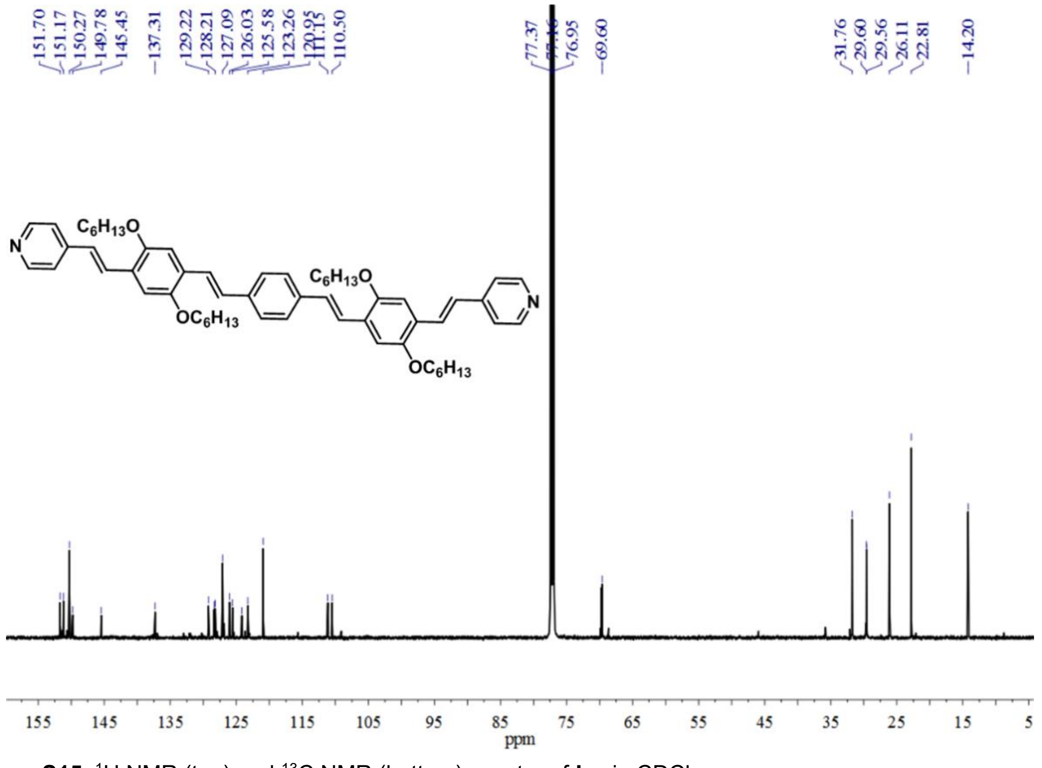

Figure S15. ^{1}H NMR (top) and ^{13}C NMR (bottom) spectra of $\mathbf{L_{2b}}$ in $CDCl_3$.

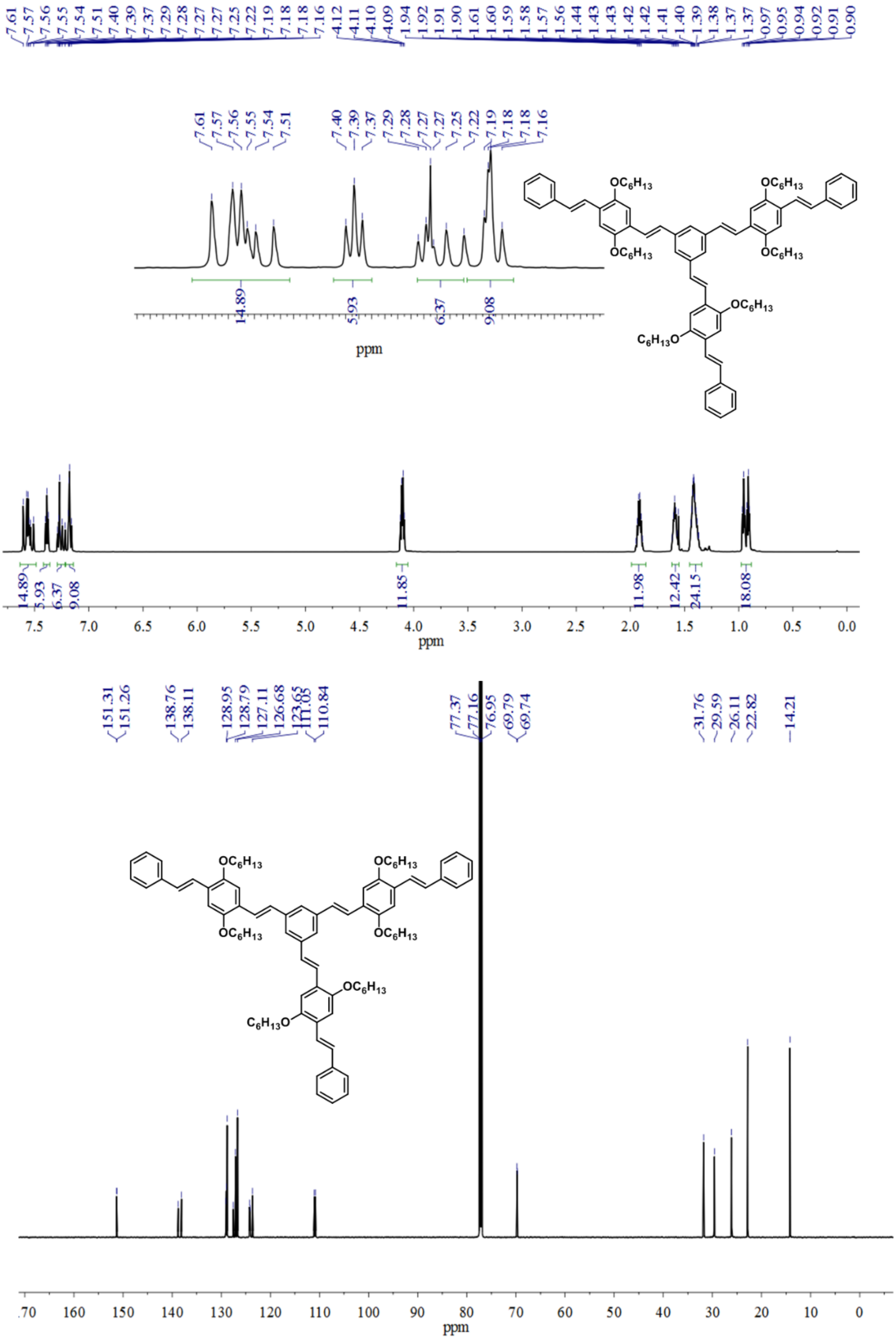

Figure S16. ^{1}H NMR (top) and ^{13}C NMR (bottom) spectra of $\mathbf{Y_0}$ in $CDCl_3$.

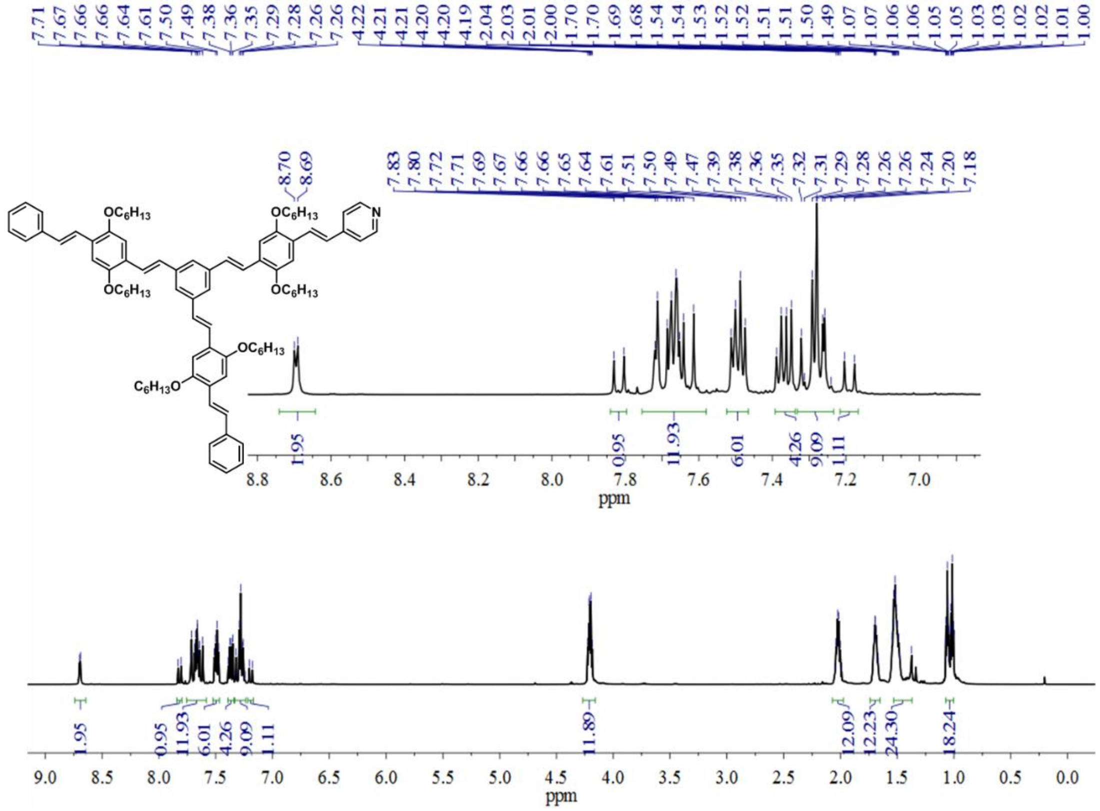

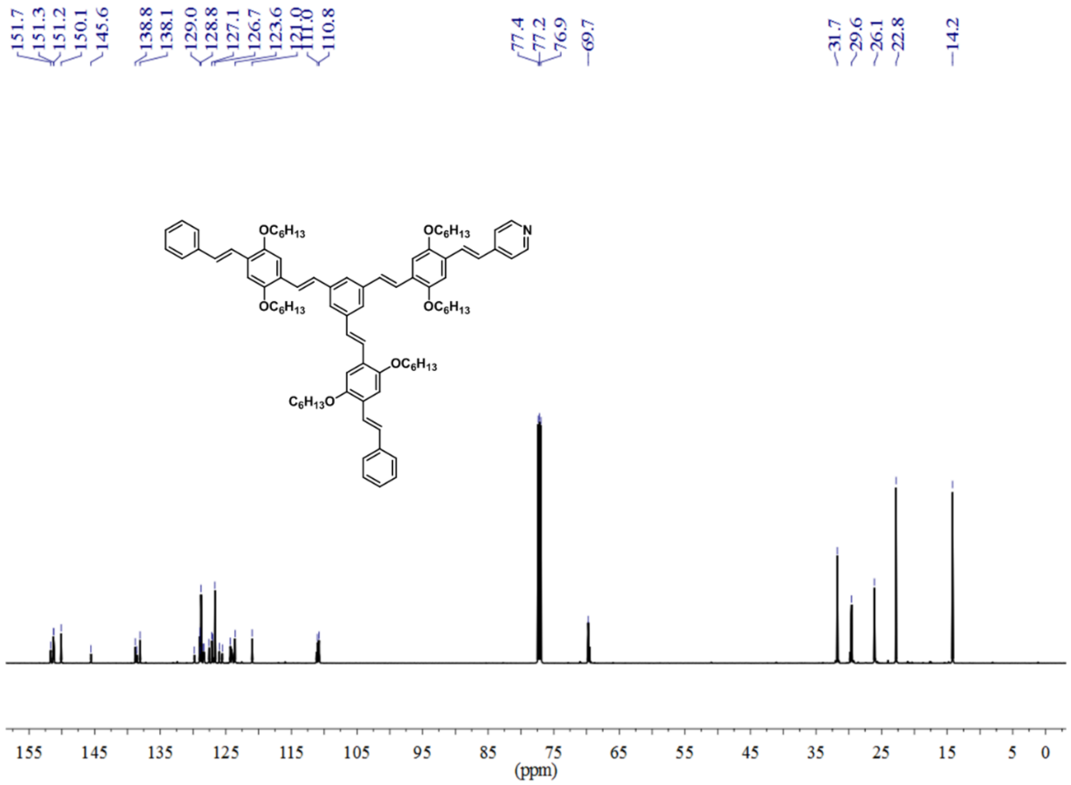

Figure S17. ^{1}H NMR (top) and ^{13}C NMR (bottom) spectra of $\mathbf{Y_1}$ in $CDCl_3$.

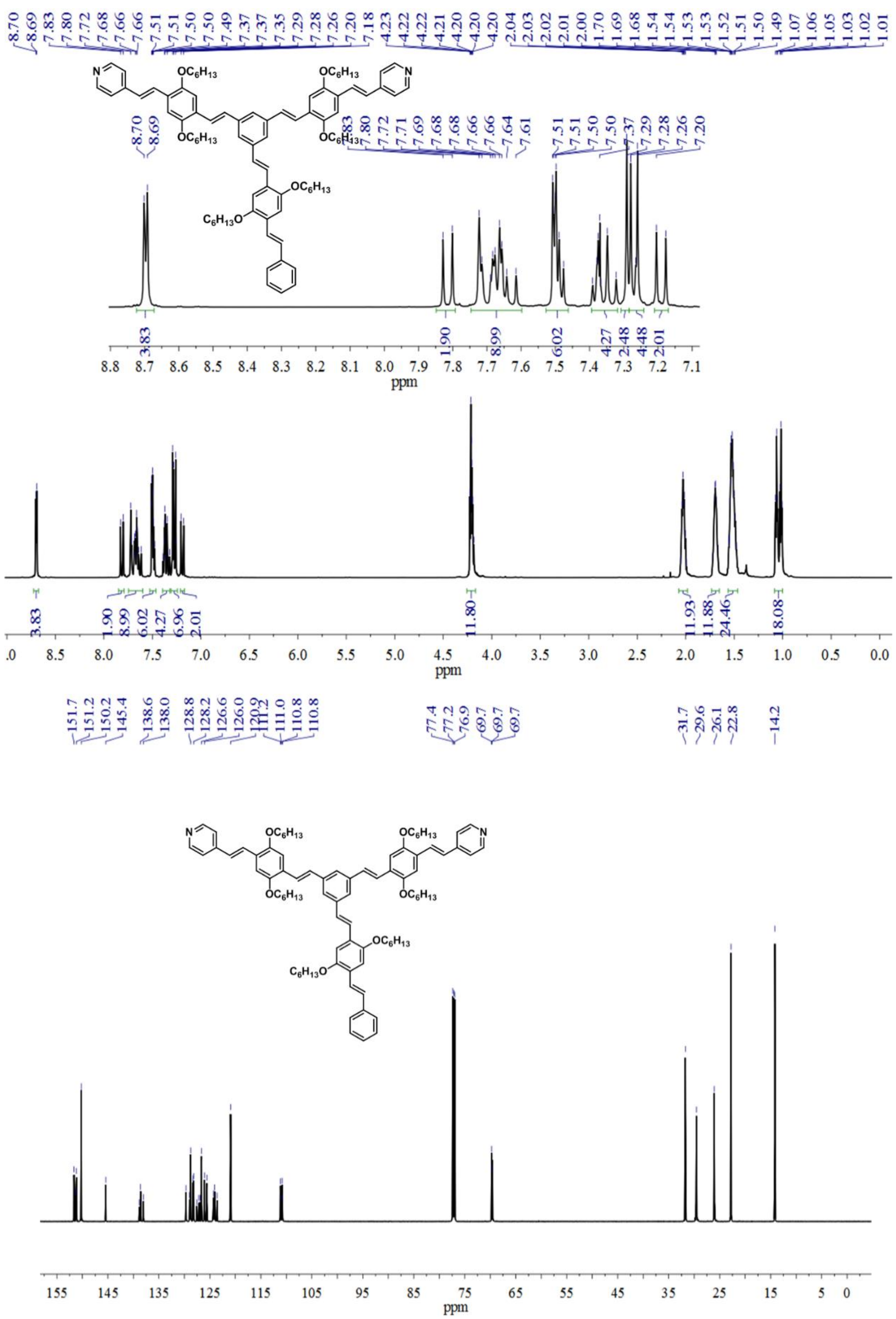

Figure S18. ^{1}H NMR (top) and ^{13}C NMR (bottom) spectra of $\mathbf{Y_2}$ in $CDCl_3$.

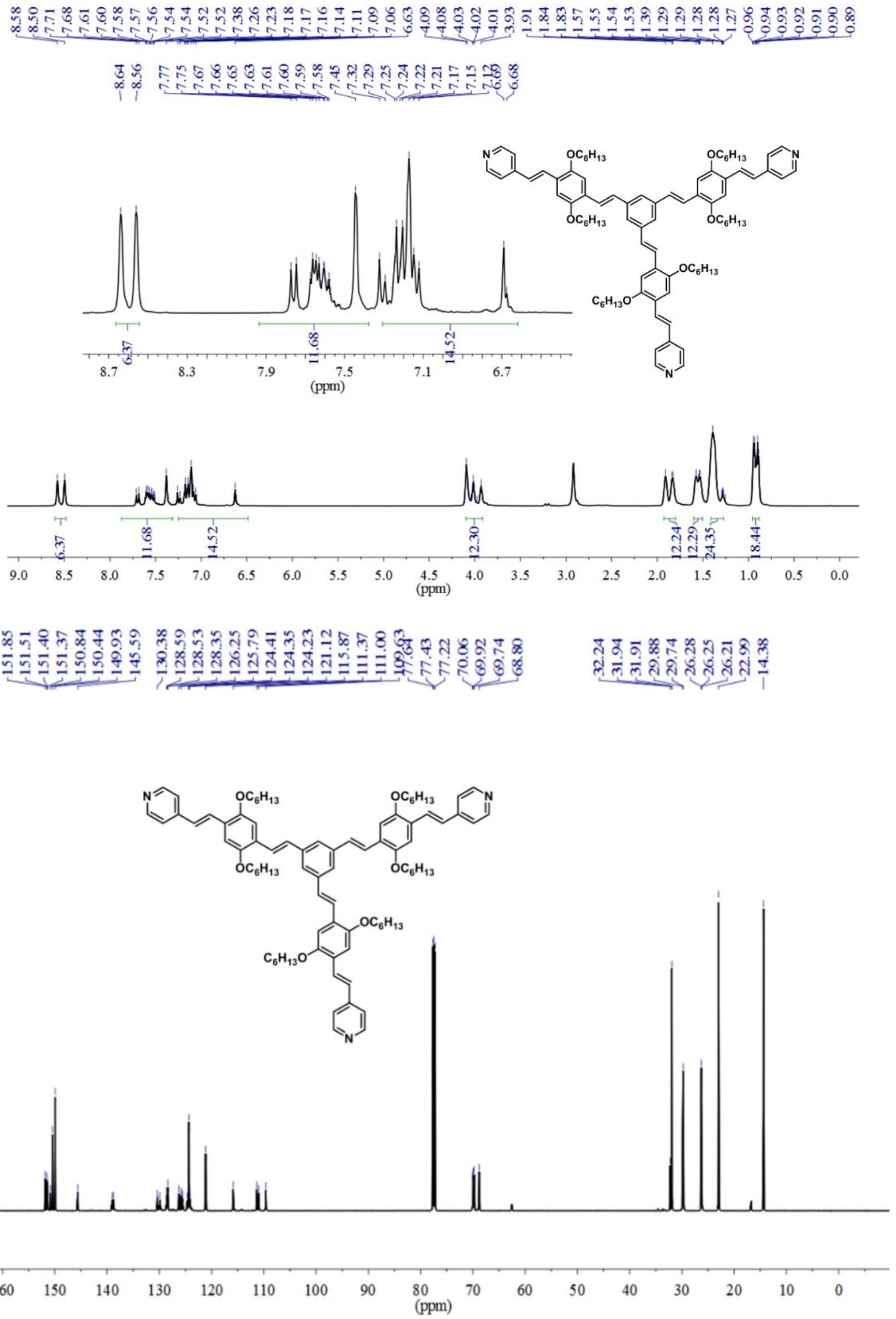

Figure S19. ^{1}H NMR (top) and ^{13}C NMR (bottom) spectra of $\mathbf{Y_3}$ in $CDCl_3$.

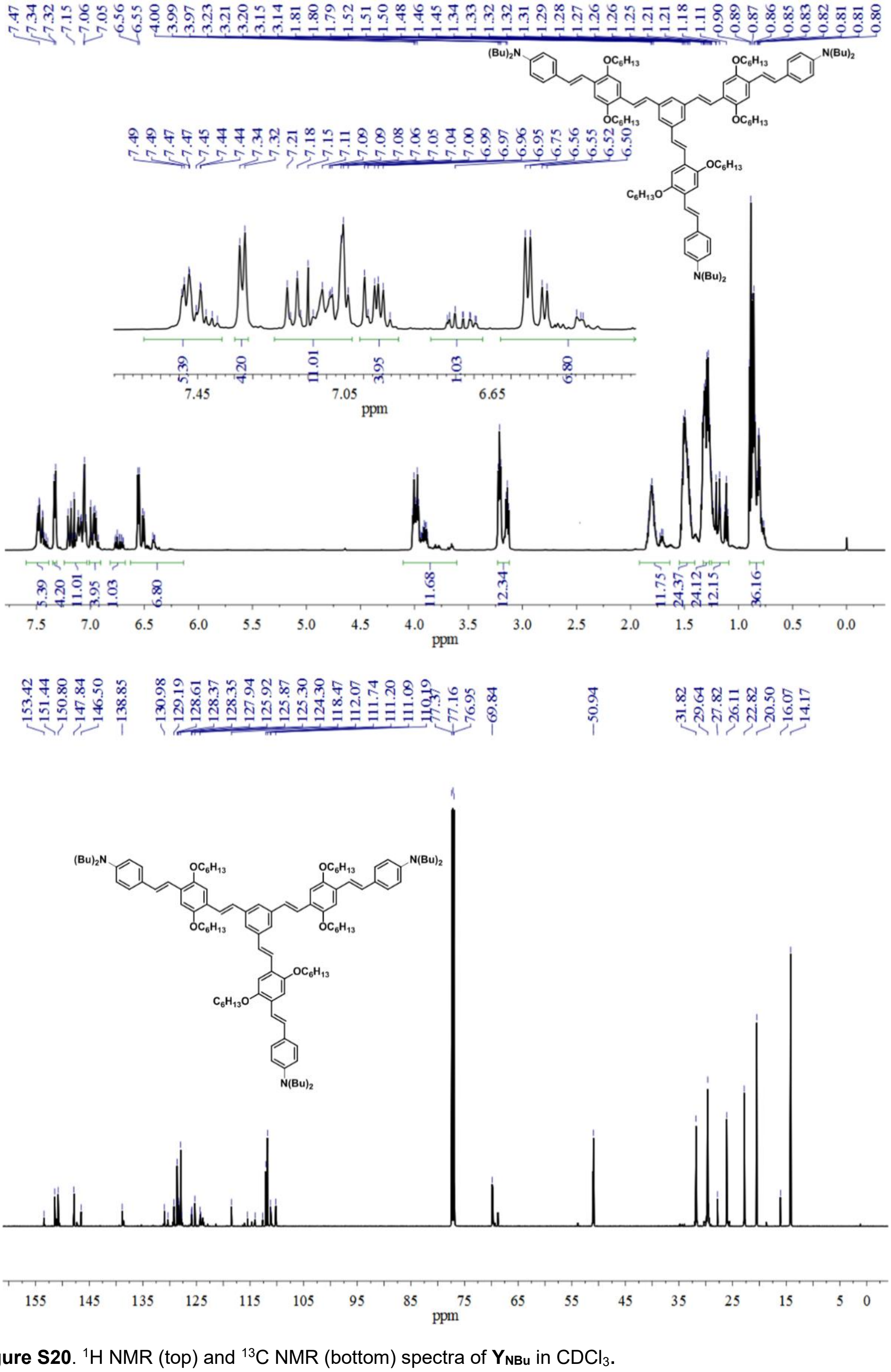

Figure S20. ^{1}H NMR (top) and ^{13}C NMR (bottom) spectra of **Y$_{NBu}$** in $CDCl_3$.

S8. References

[1] M. Nojima, K. Kosaka, M. Kato, Y. Ohta and T. Yokozawa, *Macromol. Rapid Commun.* **2016**, *37*, 79-85.

[2] C. H. Huang and Y. J. Chang, *Tetrahedron Lett.* **2014**, *55*, 4938-4942.

[3] E. W. Miller, J. Y. Lin, E. P. Frady, P. A. Steinbach, W. B. Kristan and R. Y. Tsien, *PNAS* **2012**, *109*, 2114-2119.

[4] H. Meier, M. Lehmann, H. C. Holst and D. Schwöppe, *Tetrahedron* **2004**, *60*, 6881-6888.

[5] N. Mizoshita, M. Ikai, T. Tani and S. Inagaki, *J. Am. Chem. Soc.* **2009**, *131*, 14225-14227.

[6] Z. Shi, J. Davies, S.-H. Jang, W. Kaminsky and A. K.-Y. Jen, *Chem. Commun.* **2012**, *48*, 7880-7882.
